# A high-throughput ratiometric method for imaging hypertrophic growth in cultured primary cardiac myocytes

**DOI:** 10.1016/j.yjmcc.2019.04.001

**Published:** 2019-05

**Authors:** Aminah A. Loonat, M. Kate Curtis, Mark A. Richards, Graciela Nunez-Alonso, Johanna Michl, Pawel Swietach

**Affiliations:** University of Oxford, Department of Physiology, Anatomy & Genetics, Parks Road, Oxford OX1 3PT, United Kingdom

**Keywords:** Hypertrophy, Fluorescence, Sulforhodamine B, IP_3_ signaling, GPCR, Cell culture, Drug screening

## Abstract

Maladaptive hypertrophy of cardiac myocytes increases the risk of heart failure. The underlying signaling can be triggered and interrogated in cultured neonatal ventricular myocytes (NRVMs) using sophisticated pharmacological and genetic techniques. However, the methods for quantifying cell growth are, by comparison, inadequate. The lack of quantitative, calibratable and computationally-inexpensive high-throughput technology has limited the scope for using cultured myocytes in large-scale analyses. We present a ratiometric method for quantifying the hypertrophic growth of cultured myocytes, compatible with high-throughput imaging platforms. Protein biomass was assayed from sulforhodamine B (SRB) fluorescence, and image analysis calculated the quotient of signal from extra-nuclear and nuclear regions. The former readout relates to hypertrophic growth, whereas the latter is a reference for correcting protein-independent (e.g. equipment-related) variables. This ratiometric measure, when normalized to the number of cells, provides a robust quantification of cellular hypertrophy. The method was tested by comparing the efficacy of various chemical agonists to evoke hypertrophy, and verified using independent assays (myocyte area, transcripts of markers). The method's high resolving power and wide dynamic range were confirmed by the ability to generate concentration-response curves, track the time-course of hypertrophic responses with fine temporal resolution, describe drug/agonist interactions, and screen for novel anti-hypertrophic agents. The method can be implemented as an end-point in protocols investigating hypertrophy, and is compatible with automated plate-reader platforms for generating high-throughput data, thereby reducing investigator-bias. Finally, the computationally-minimal workflow required for obtaining measurements makes the method simple to implement in most laboratories.

## Highlights

• Maladaptive hypertrophy of myocytes can lead to heart failure.
• Common methods for tracking growth in cultured myocytes are inadequate.
• We design and test a method for tracking myocyte hypertrophy in vitro.
• The method provides a ratiometric index of growth for high throughput analyses.
• Using the method, we characterize further details of (anti)hypertrophic responses.

## 1 Introduction

Cardiac myocytes can undergo hypertrophic growth in response to increased work-load. This can be physiological in response to regular exercise or pregnancy, or pathophysiological in conditions of cardiovascular disease such as ischemia, hypertension or cardiomyopathy [1]. Whilst physiological hypertrophy is reversible and leads to enhanced function, pathological hypertrophy can lead to arrhythmias, heart failure and/or sudden death [2,3]. Since hypertrophy is associated with an altered program of gene expression [4], controlled by transcription factors that respond to neuro-hormonal stimuli [[4], [5], [6]], a routine method for investigating pro-hypertrophic cascades is to apply such chemical triggers to myocytes in vivo using delivery devices [[7], [8], [9]], or in vitro by tissue culture methods [10,11]. Whilst cardiac hypertrophy triggered in experimental animals produces growth in the context of an intact circulation and subject to relevant adaptive or compensatory influences, it has disadvantages, namely the inability to generate high-throughput data, limited compliance with methods for interrogating the molecular detail of cascades, difficulties in controlling and monitoring the cellular environment, as well as practical and ethical restrictions on permissible experimental maneuvers. To complement and support the findings drawn from animals, cultured primary myocytes, such as neonatal rat ventricular myocytes (NRVMs), are routinely used in cardiac laboratories to address the shortcomings of in vivo experiments [12]. In comparison with isolated adult ventricular myocytes, NRVMs can survive in culture for extended periods of time, which is necessary for tracking hypertrophy.

Various readouts have been trialed to gauge the outcomes of hypertrophy under culture conditions, including cell dimensions, protein biomass, and molecular markers. Cell volume can be assayed in re-suspended myocytes using the Coulter principle, but the process of dissociating cells from their substrate can be damaging and may introduce artefacts, such as hypercontraction. The area occupied by cells on a coverslip is intuitively close to being a direct assay of growth [[9], [10], [11],^13^,^14^]; however, there are multiple practical issues. When constructing the outline of cells for measurements, this approach ignores growth in cross-sectional area, an early hypertrophic response [15]. Furthermore, outlines of individual cells are difficult to resolve in monolayers that are confluent, a necessary condition for hosting realistic interactions within myocyte networks. Geometric measurements, albeit very popular, are tedious, yield low-throughput data, and may inadvertently introduce investigator-bias. Noteworthy efforts have been made to automate cell size measurements using computationally-intensive image-processing algorithms [[16], [17], [18]], but issues arising from variations in cell height, cell-on-cell overlap, and permissible upper limits on confluency remain. Markers of hypertrophy, such as atrial and brain natriuretic peptides, α-skeletal muscle actin and β-myosin heavy chain, can be assayed by immunoreactivity or from message level [9,[19], [20], [21], [22]]. However, these readings often lack adequate quantitative resolving power, not least because of their non-linear and marker-specific relationship with cellular growth.

Analyses of protein biomass, such as the incorporation of labelled amino acids [13,23,24] or staining with bicinchoninic acid (BCA) [19] or sulforhodamine B (SRB) [25] are less user-intensive and avoid issues that could arise from subjective choices of measurement criteria. However, contemporary protocols lack appropriate and concurrently-registered reference markers to correct for cell number and protein-independent variables, such as light path for dye-based assays. Without such corrections, it is not possible to compare results between experiments, let alone between different laboratories. Furthermore, some methods for quantifying protein biomass require adequate cellular bulk which may not be compatible with high-throughput plate-based experiments.

Here, we describe a method for measuring NRVM hypertrophy adapted for high-throughput analyses in plate-based imaging systems. Our method uses the protein-binding aminoxanthene dye SRB [26] and simple image processing to segregate the fluorescence signal into nuclear and extra-nuclear components, based on the pattern of nucleic acid-binding dyes (e.g. Hoechst-33,342). The ratio of extra-nuclear to nuclear SRB signal, normalized to the number of cells, yields a ratiometric readout of growth. The merit of our method was demonstrated by its ability to provide a more complete pharmacological characterization of agonist-induced hypertrophy, and screen agents for anti-hypertrophic actions.

## 2 Methods

### 2.1 Cell isolation and culture

All animals were euthanized by cervical dislocation according to Schedule 1 of the Animals (Scientific Procedures) Acts 1986. All animal experiments have been approved by Oxford University ethical review boards and conform to the guidelines from Directive 2010/63/EU. Primary neonatal rat ventricular myocytes were obtained from 1 to 2 day old Sprague-Dawley rats. Cells were isolated from ventricular tissue by enzymatic digestion [13], and a ‘pre-plating’ step was introduced to extract fibroblasts from the myocyte-enriched supernatant (see *Supplement*). Unless indicated otherwise, seeding density was 60,000 cells per 200 μL well, which normally yields 2000–3500 cells per field of view imaged by the Cytation platform. Adult myocytes were isolated from Langendorff-perfused heart from 10 to 12 week old rats or 7–9 week old mice, using a previously published method [27]. Freshly-isolated calcium-tolerate cells were used for experiment on the day of the isolation.

### 2.2 Microscopy

Cells plated in Ibidi chambers were imaged on a Leica SP5 or Zeiss LSM 600 confocal system with a ×40 objective and sequential excitation at 355 nm (Hoechst-33,342) and 514 nm (SRB). Fluorescence was measured through an open pinhole at 400–440 nm and 620–660 nm. Cells cultured in 96-well plates were imaged in a Cytation 5 (Biotek) system with a dry ×4 objective and sequential excitation at 377 nm and 531 nm. Fluorescence was measured at 417–477 nm and 630–650 nm.

### 2.3 Image analysis

Images of Hoechst-stained nuclei were thresholded (Ridler and Calvard method) to produce a binary mask. Hoechst-positive particles were analyzed for geometry to obtain an estimate of the number of mononucleated myocytes, binucleated myocytes, and fibroblasts, as described later. Pixels of the SRB image, after background subtraction, were segregated into those representing nuclear regions and extra-nuclear regions (nSRB, eSRB).

### 2.4 Statistics

T-tests were performed between data obtained from independent samples. One-way ANOVA with hierarchal analysis was performed for data that included technical repeats [28]. Number of observations are presented as “(number of wells or cells/number of independent isolations each obtained from 10-24 rat pups)”.

See *Supplement* for details of cell culture, imaging, immunofluorescence and real-time PCR.

## 3 Results

### 3.1 Acquiring SRB fluorescence as a measure of cell size

The protein-binding probe SRB is used extensively in cytotoxicity screens [26,29] because it provides a stoichiometric measure of protein content and can be adapted for use in conventional imaging microscopes and automated plate readers [30]. Linearity of the SRB signal over a range of cell confluency from ~1% to 200%, and cost-effectiveness have been demonstrated in a number of cancer studies [26]. The build-up of protein biomass during hypertrophic growth is expected to produce a stronger SRB fluorescence signal. A final step in conventional SRB-based cytotoxicity protocols [26,29] is to dissociate SRB from protein (using Tris-base), and then take optical density measurements as the readout of protein biomass. However, this method cannot accurately determine cell size because it is not readily corrected for extraneous, growth-independent factors (e.g. imaging settings) and the cell count. To preserve information relating to the subcellular distribution of SRB fluorescence *in situ* and the number of cells, the standard protocol was modified to omit the final alkaline wash step. To visualize nuclear areas, fixed (4% paraformaldehyde, 10 min) and permeabilized (0.5% Triton X-100, 10 min) myocytes were stained with the nuclear dye Hoechst-33342 dissolved in PBS (10 μg/mL, 10 min) before staining with SRB dissolved in 1% acetic acid. This particular order of staining was necessary because SRB is only able to bind to amino acid residues under mildly acidic conditions, whereas Hoechst staining is best near neutral pH. Indeed, if the staining order were reversed, SRB would dissociate from proteins at the pH of PBS.

**Fig. 1:**
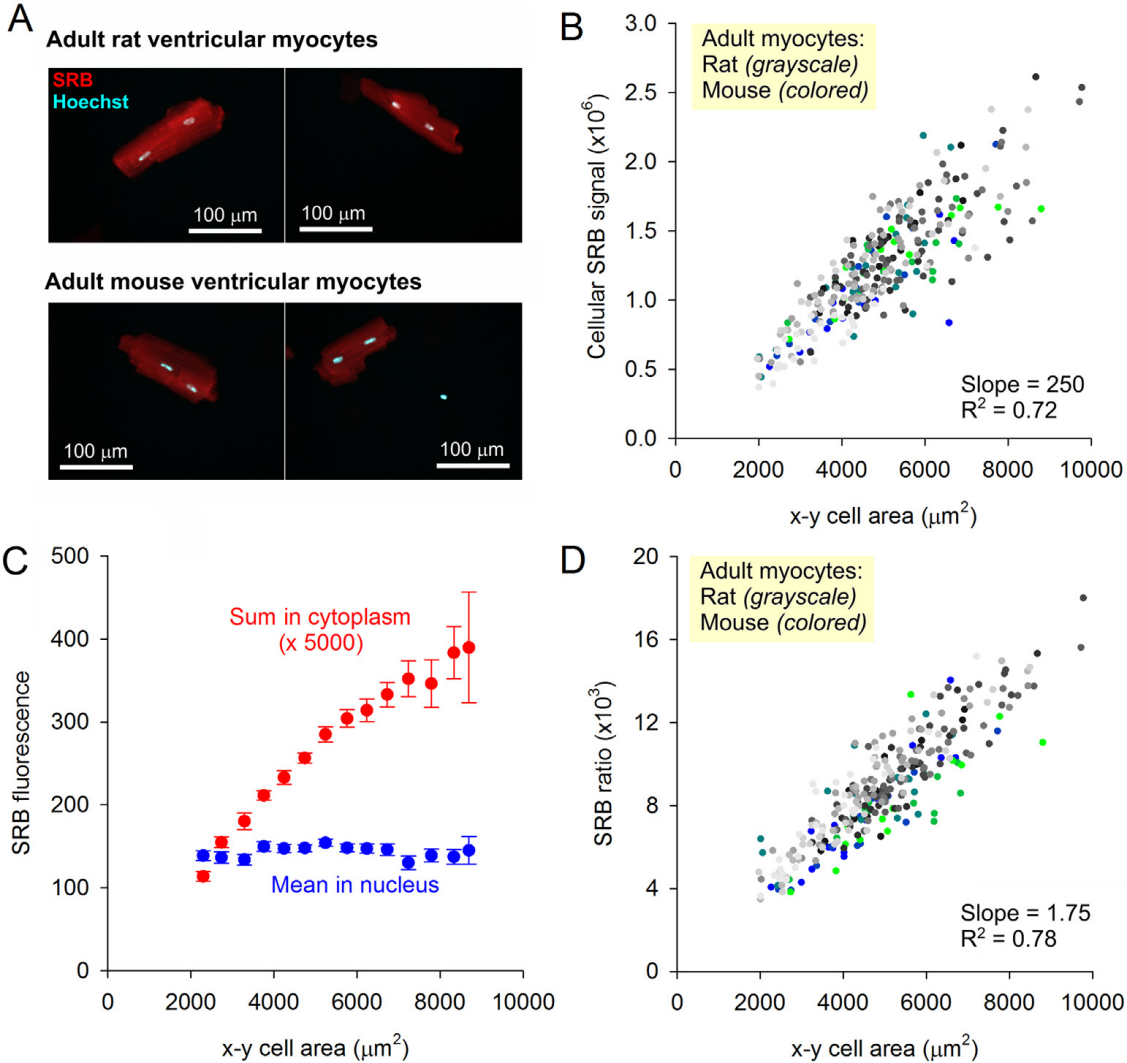
Staining adult ventricular myocytes with SRB. (A) Fluorescence image of rat and mouse ventricular myocytes stained with SRB and Hoechst 33342, and excited sequentially on a confocal system with open pinhole. (B) Relationship between total SRB fluorescence collected within the cell outline (a measure of volume) and cell area in the x-y plane (i.e. a measure that ignores cell thickness in the z-direction). Each color/shade denotes data from a separate batch of cells: grayscale represent rat myocytes (*N* = 221, 10 batches); colors represent mouse myocytes (*N* = 73, 5 batches). (C) Total SRB fluorescence in the non-nuclear cytoplasmic area (red) rises near-linearly with cell area, whereas mean SRB signal in nuclear areas is independent of cell size, making the latter a suitable reference for ratiometric analyses (mean ± SEM; N-5-52 cells per bin) (D) Ratio of extra-nuclear to nuclear SRB signal, plotted as a function of cell area. This ratiometric analysis improves correlation (Pearson coefficient R^2^) and corrects for batch-to-batch differences in extraneous variables such as imaging settings. (For interpretation of the references to color in this figure legend, the reader is referred to the web version of this article.)

SRB binding to cardiac protein biomass was first measured in adult ventricular myocytes (Fig. 1A) and quantified as a function of myocyte area (Fig. 1B). To allow for adequate Hoechst staining quality, optimal SRB concentration was determined to be 0.004%. Since Hoechst fluorescence progressively declines in the acidic environment required for SRB binding [31], staining with SRB was restricted to 10 min. Slides were then dried and imaged for Hoechst and SRB fluorescence by sequential excitation. This staining protocol was performed on 10 batches of rat myocytes and 5 batches of mouse myocytes using the same imaging settings (each batch is denoted by a different color in Fig. 1B/D). Images were obtained from a large number of cells to capture the natural variation in cell size among wild-type myocytes (mean ± SD length of rat and mouse myocytes was 132.7 ± 22.9 μm and 128.9 ± 26.7 μm, respectively).

Fluorescence excited across the thickness of cells and collected across the field-of-view provides a readout that is proportional to the total protein content within the imaged area. SRB fluorescence correlated strongly with cell area (Pearson's R^2^ = 0.72). However, this SRB signal can be influenced by biomass-independent variables, such as light-path or equipment settings, or even variation in the execution of staining protocol. A suitable reference for SRB fluorescence is the signal from nuclear regions, which can be visualized with Hoechst or DAPI stains. Nuclear-derived SRB fluorescence was found to be independent of cell size (Fig. 1C), making it suitable as a reference point for normalizing cytoplasmic SRB signal. Thus, it is possible to obtain a ratiometric index of myocyte size from the total cytoplasmic SRB signal and mean nuclear SRB fluorescence (Fig. 1D), adopting a familiar approach used with many ion-sensing fluorescent probes [32]. Such an index retains good proportionality to cell size, and is independent of extraneous factors, such as imaging settings. Some variation in the relationship plotted in Fig. 1D will arise from differences in cell height, which is captured by SRB fluorescence but not by measurements of cell area in the x-y plane. Indeed, growth in cell-height likely explains why the relationship in Fig. 1C becomes sublinear for large cells.

### 3.2 Normalizing SRB fluorescence to cell count

**Fig. 2:**
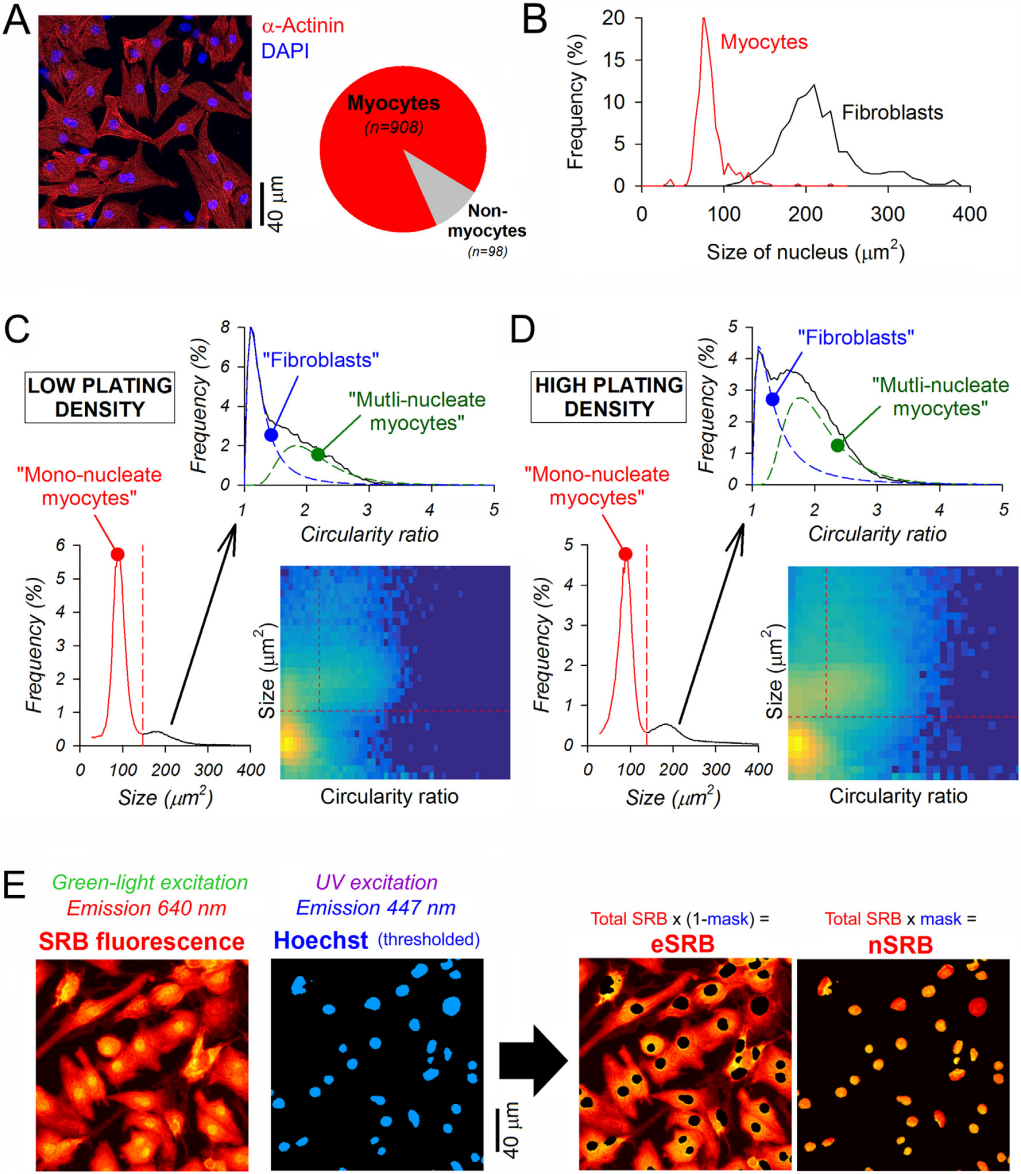
Workflow for determining counts of cells by sub-type and the subcellular distribution of SRB. (A) Immunofluorescence confocal image of NRVM monolayer stained for α-actinin (cardiomyocyte marker) and the nuclear stain DAPI. Based on an analysis of images from 14 cell isolations, ~90% of cells were identified as myocytes. (B) Statistical distribution of the area of Hoechst-positive particles detected in myocytes under normal NRVM culture, compared to fibroblast-enriched cultures obtained from the pre-plating step and imaged in separate experiments (from 4 isolations). Data obtained by high-magnification microscopy. (C) Analysis of nuclear particle geometry in NRVMs cultured at low density (~1500 particles/field of view); data pooled from 96 wells imaged by Cytation 5. Circularity ratio is the quotient of the long-axis to short-axis diameter of the particle. Analyzed by area, the majority of particles are representative of mononucleated myocytes. When plotted against circularity ratio, the population with larger nuclei can be described as the sum of two log-normal distributions representing fibroblasts and binucleated myocytes (dashed lines). (D) Analysis repeated on cells cultured at higher density (~3000 particles per field of view). (E) Sequential imaging of SRB and Hoechst. A binary mask, determined from the pattern of Hoechst staining, was used to segregate extra-nuclear and nuclear SRB signals (eSRB, nSRB).

When imaging larger populations of cells, such as a monolayer of NRVMs, the total SRB signal collected will also depend on the number of cells. A cell count could be derived from an analysis of Hoechst-positive particles. To test the most appropriate analysis strategy, NRVMs were isolated from pups, and a pre-plating step separated the majority of non-myocytes cells, including fibroblasts. The myocyte-enriched supernatant was then plated on coverslips at a seeding density that is permissive for establishing network behavior critically important for physiological signaling. An example of a monolayer is shown in Fig. 2A. In this experiment, myocytes were identified positively by immunoreactivity to α-actinin and the nuclei were visualized with DAPI. Based on an analysis from 14 isolations, the percentage of myocytes was consistently at 90%, i.e. most of the SRB signal collected across a field of view would be expected to be myocyte in origin. A determination of the fibroblast count would be desirable for quality-control purposes, particularly if test-treatments are expected to influence proliferation. This could be implemented in the protocol by including an immunofluorescence staining step for a suitable fibroblast marker, but a much simpler approach is to analyze nuclei geometry on the basis that non-dividing cardiac fibroblasts have significantly larger nuclei than myocytes. This was demonstrated by analyzing nuclei of myocytes identified by α-actinin staining, and nuclei of fibroblasts maintained separately in culture following the ‘preplating’ step (Fig. 2B). However, nuclear size alone cannot distinguish fibroblasts from binucleate myocytes, which are found in NRVM cultures (~10% of the myocyte population [33]). An additional gating criterion relating to the shape of Hoechst-positive particles is required to distinguish these two types of cells. A binucleate myocyte is apparent from its elongated Hoechst staining, or closely abutting Hoechst particles. This can be quantified by a high (≫1) long-axis to short-axis diameter ratio (circularity ratio). In contrast, fibroblast nuclei would appear more circular. This two-way analysis was performed on NVRM cultures seeded at low (~1500 particles/field of view; Fig. 2C) or high density (~3000 particles/field of view; Fig. 2D), and imaged on a high-throughput platform. Two-dimensional histograms of Hoechst particle analysis identified the size cut-off for mono-nucleated myocytes. The population representing larger nuclei was separated into two sub-populations based on circularity ratio using Gaussian (log-normal) mixture modelling. Thus, the distribution of cells among the three groups can be obtained for each experiment. Based on an analysis of cells obtained from 21 isolations aliquoted into a total of 300 wells and cultured in control media, 77% (SD 12%) of cells were mono-nucleate myocytes, 16% (SD 14%) were binucleate myocytes, and the remaining 7% (SD 3%) were fibroblasts.

Thresholding of Hoechst fluorescence generated a binary mask for segregating the monolayer-wide SRB signal into its nuclear and extra-nuclear components (nSRB, eSRB). Fig. 2E shows a representative, high-magnification fluorescence image of a monolayer of NRVMs, acquired on a confocal system through an open pinhole. Since the field-averaged eSRB signal includes both cytoplasm and background areas, it will increase as cells expand over their growth substrate. Columns identified as nuclear areas will include a sliver of cytoplasm above and below the nuclear mass that would not be included in the nominally extra-nuclear SRB signal. However, for a cell density of ~2000 particles per field of view, nuclear regions occupy ~7% of the area, and since cytoplasm in these regions occupies a small fractional volume along the z-axis, the underestimate in eSRB will be considerably less than 5%, and thus can be ignored.

In contrast to growth-dependent eSRB, mean nSRB fluorescence is insensitive to hypertrophy (Fig. 1C) and therefore a suitable reference marker to the eSRB signal. Hypertrophy can thus be quantified ratiometrically from the eSRB signal across the field of view, nSRB averaged for all nuclear particles, and the number of cells:

(1) $$\text{Ratiometric}\;\mathrm{SRB}\;\text{index}=\frac{\text{eSRB signal}}{\text{mean nSRB signal}\times \text{number of cells}}$$

In this normalization, nuclei identified as binucleate (i.e. abutting or in close proximity) are counted as belonging to a single cell. Since myocytes outnumber fibroblasts by an order of magnitude, the vast majority of the SRB signal is myocyte-derived and no further correction factors were deemed necessary.

### 3.3 Obtaining a SRB-derived ratiometric quantification of cell growth

**Fig. 3:**
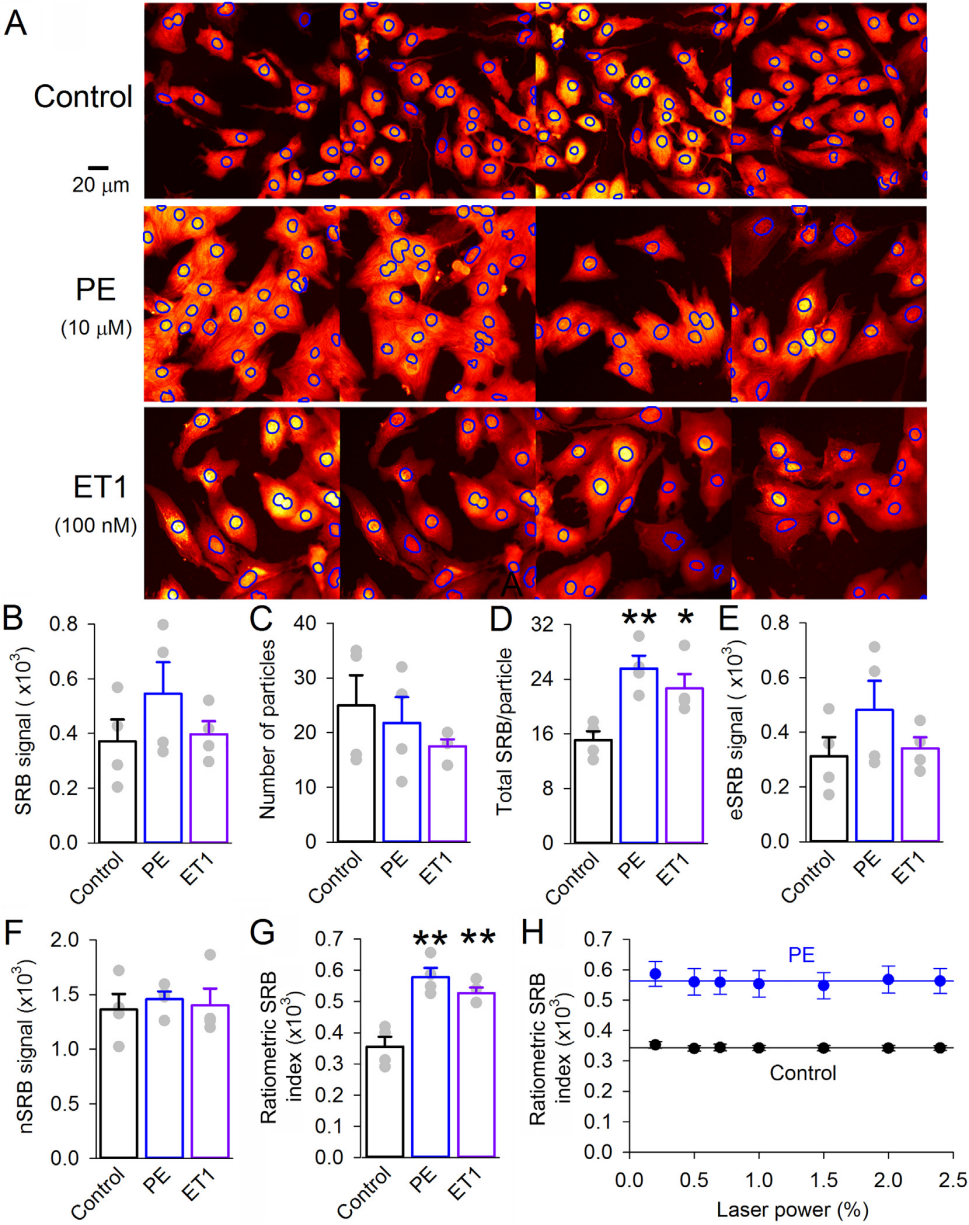
Obtaining a ratiometric SRB index of hypertrophy in NRVMs. (A) NRVM monolayer imaged for SRB fluorescence under x40 magnification. Nuclear regions, identified from Hoechst staining, outlined in blue. Monolayers were cultured in 10 μM phenylephrine (PE; stabilized with 100 μM ascorbate) or 100 nM endothelin-1 (ET1) to trigger hypertrophy. Images were taken in randomly selected fields of view. (B) Total SRB fluorescence and (C) number of nuclei in a given field of view give (D) ratio showing significant growth in PE and ET1; however, this readout is influenced by protein-independent variables, e.g. equipment-related settings (n = 4 images). (E) Extra-nuclear SRB (eSRB) and (F) nuclear SRB (nSRB) signal. (G) Ratiometric index of hypertrophy calculated by Eq. (1). Note the spread of data is greatly reduced by the ratiometric approach. (H) Changing laser power does not affect the ratiometric SRB index. Experiments performed on cells obtained from an isolation of 10–24 rat pups, plated into four chambers treated independently; * P < 0.05; ** P < 0.01; *t*-test (two-sided). Mean ± SEM. (For interpretation of the references to color in this figure legend, the reader is referred to the web version of this article.)

To test the relationship between SRB signal and cell growth, hypertrophic responses in NRVMs were triggered by chemical stimuli that evoke inositol trisphosphate (IP_3_) signaling: (i) the α_1_-receptor agonist phenylephrine (PE; 10 μM) [34] supplemented with ascorbate to minimize oxidative degradation, or (ii) endothelin-1 (ET1; 100 nM) [35] for 48 h. At the start of chemical stimulation (including its time-matched controls), media were replaced with fresh aliquots that had been pre-equilibrated in the 5% CO_2_ incubator for at least 1 h. Monolayers were imaged on a confocal system through an open pinhole and a x40 objective. Relative to agonist-free controls, cells appeared visibly hypertrophied in response to PE or ET1 (Fig. 3A). Total SRB signal (Fig. 3B) was unable to resolve differences in growth, but when normalized to the number of particles (Fig. 3C), the hypertrophic effect of agonists became apparent (Fig. 3D). This fluorescence readout is, however, influenced by extraneous variables, such as the imaging settings (e.g. stronger laser power would erroneously give a higher reading). To correct for such factors, the SRB signal was segregated into its extra-nuclear (eSRB; Fig. 3E) and nuclear (nSRB; Fig. 3F) components, and converted to a ratio as defined by Eq. (1) (Fig. 3G). This index is insensitive to changes in imaging settings; for example, doubling laser power would raise eSRB and nSRB by the same factor, but not affect their ratio. This was confirmed experimentally (Fig. 3H), by demonstrating the constancy of ratio when the 555 nm laser power was varied over an order of magnitude.

### 3.4 Up-scaling the ratiometric method for use with high-throughput platforms

**Fig. 4:**
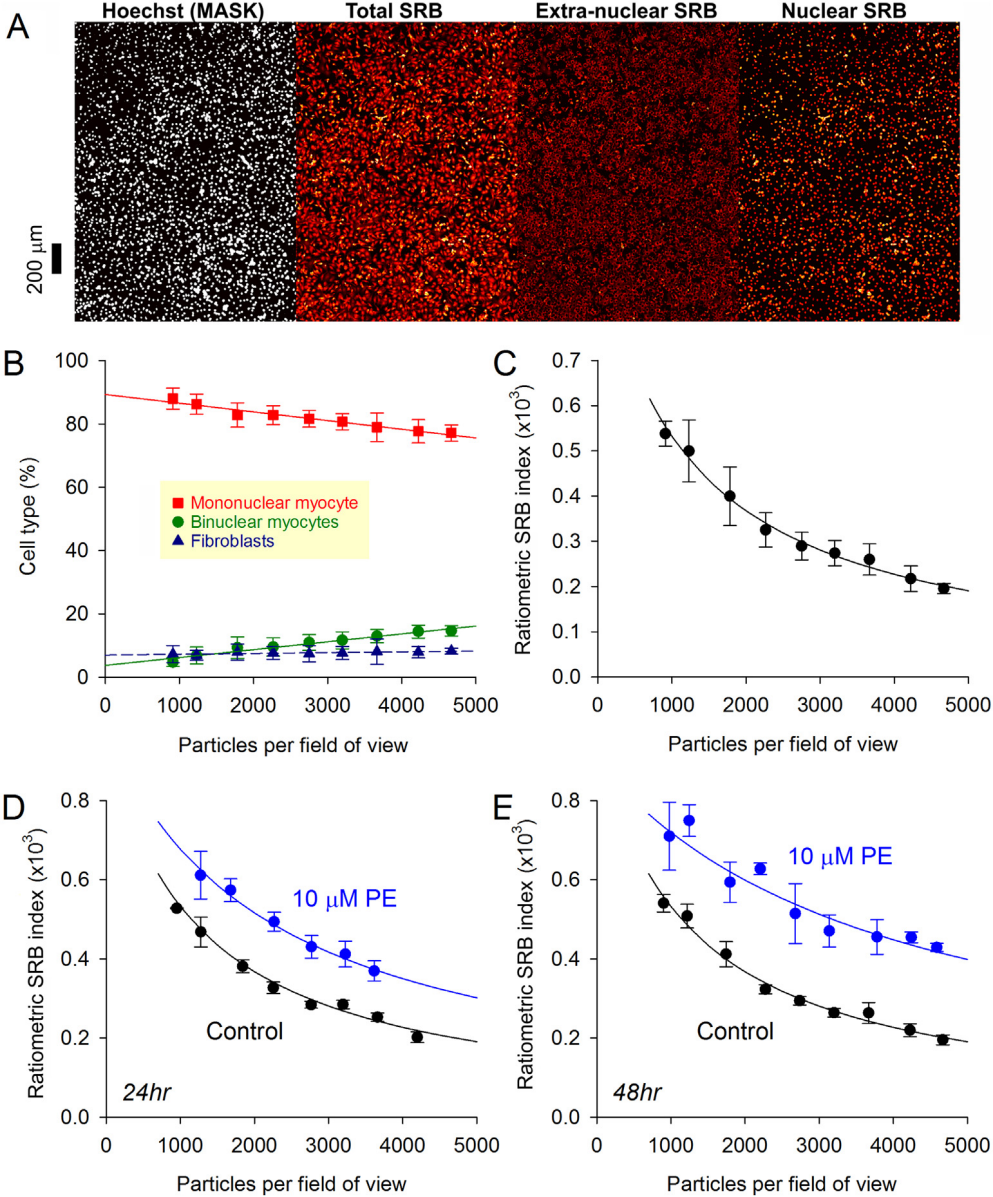
Scaling-up the SRB method for high-throughput analyses. (A) Imaging NRVM monolayers on an automated plate-reader system. Hoechst staining produces binary mask for identifying nuclear regions and segregating SRB signal into nuclear and extra-nuclear components (nSRB, eSRB) (n = 12/3). (B) Allocation of nuclei by cell type (fibroblast, mono- or binucleated myocyte) based on an analysis of Hoechst-positive particle geometry. Mean ± SD. (C) Under control culture conditions, ratiometric SRB index of cell growth decreases as cell density increases. Mean ± SD. Best fit: exponential. (D) Effect of 24 h and (E) 48 h PE stimulus on ratiometric SRB index relative to time-matched controls, plotted as a function of cell density. Mean ± SEM.

Plate readers with low-power objectives can capture SRB across a larger field-of-view and in an automated manner compatible with high-throughput analyses. Imaging a larger area of a monolayer also eliminates artefacts, which may be introduced by bias in selecting a field-of-view under higher magnification. To test if the SRB-based method is suitable for such up-scaling, NRVM monolayers were grown in 96-well plates at various seeding densities. Fig. 4A shows fluorescence images from an exemplar monolayer, showing the nuclear mask and the SRB signal segregated into its extra-nuclear and nuclear components. The nuclear staining pattern was analyzed to identify mono- and binucleated myocytes, and fibroblasts (Fig. 4B). The fibroblast count was 8% and appeared independent of seeding density. The myocyte fraction, in contrast, became modestly enriched in mono-nucleated cells as seeding density was reduced. The ratiometric SRB index of growth increased by a factor of >2 as density decreased from ~4500 to ~1000 (Fig. 4C), which may be explained by the increase in the space available for cell expansion. The growth response to 10 μM PE was modestly sensitive to seeding density, producing the greatest relative change at higher cell densities (Fig. 4D/E). Further experiments were performed with seeding density in the range 2000–3500 per field-of-view, which corresponded with 60,000 cells per well of a 96-well plate.

**Fig. 5:**
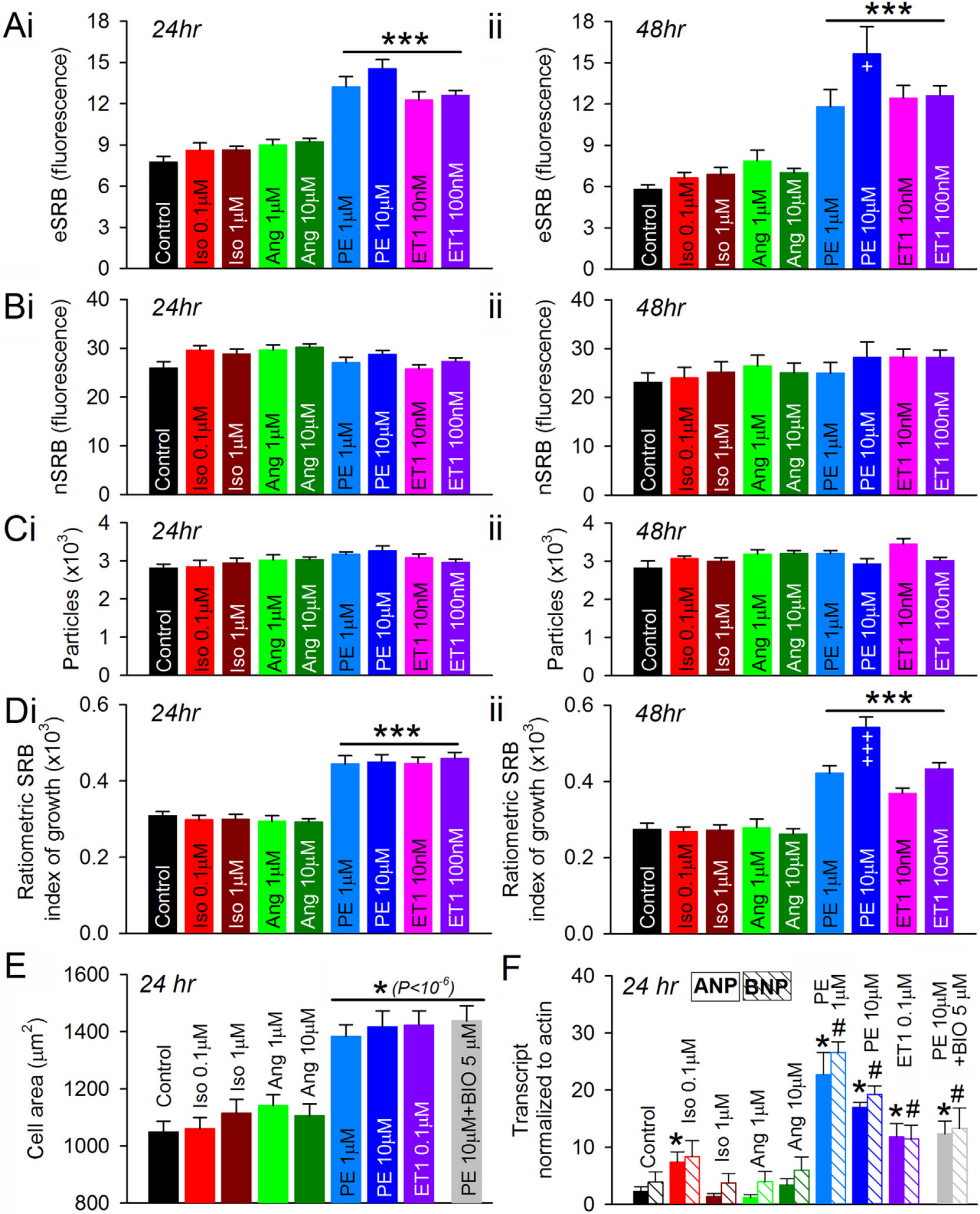
Testing agonists for pro-hypertrophic actions on NRVMs. (A) Effect of chemical agonists on eSRB and (B) nSRB fluorescence following (i) 24 or (ii) 48 h treatment (n = 12/3). (C) Hoechst-positive particle count. (D) Effects of PE and ET1 on ratiometric SRB index. Experiments performed on cells from three isolations of 10–24 rat pups (four technical repeats). Significance determined by hierarchal ANOVA (see Table S1); *** significant response relative to control; +++ significant difference relative to the lower dose of agonist. (E) Cell area from outline at sparse seeding density; myocytes visualized from immunofluorescence staining for α-actinin. As a positive control, the GSK3 inhibitor BIO was combined with PE. Recordings from 45 to 80 cells obtained from three independent isolations of 10–24 rat pups each, with four technical repeats each. Significance (vs control) determined by *t*-test with Bonferroni correction. (F) Transcripts for atrial and brain natriuretic peptides (ANP, BNP), normalized to β-actin, collected after 24 h of treatment with chemical agonists. * denotes significant change in ANP and ^#^ denotes significant change in BNP (n = 4/4; P < 0.05; *t*-test). Data obtained from lysates collected from four independent isolations of 10–24 rat pups each. Mean ± SEM.

The SRB assay was tested for its ability to resolve pro-hypertrophic effects of various agonists. Monolayers were treated with isoproterenol (Iso; 0.1 μM or 1 μM) [34], angiotensin II (Ang; 0.1 μM or 1 μM) [35], PE (1 μM or 10 μM) and ET1 (10 nM or 100 nM) for either 24 h or 48 h. In cardiac myocytes, the β-receptor agonist Iso evokes cyclic adenosine monophosphate (cAMP) signals [36], whereas the agonists PE and ET1 mobilize IP_3_ cascades [37,38]. Ang is known to be pro-hypertrophic, but this effect is mediated by fibroblasts through paracrine signaling [39]; consequently, Ang is not expected to affect growth in cultures consisting predominantly of myocytes. The data collected from five different plates are shown in Fig. 5A–D. The most pronounced agonist-related differences in SRB signal were detected in extra-nuclear regions (Fig. 5A), consistent with the bulk of cellular growth occuring outside the nucleus. In contrast, nSRB remained unchanged under all test conditions (Fig. 5B), confirming its suitability as a reference marker for ratiometric analysis.

Consistent with results obtained using high-power imaging (Fig. 3), PE and ET1 evoked a strong hypertrophic response, as measured by the ratiometric index of growth (Fig. 5D). The high resolving power of the method is apparent from the small standard error of the mean and the magnitude of statistical significance of the PE and ET1 responses (Table S1).

To validate the ratiometric SRB index as a measure of hypertrophy, its results were compared against standard assays. As a positive control, PE was combined with the GSK3 inhibitor BIO [40]. NRVM monolayers were subject to treatment with Iso, Ang, PE or ET1, and their size was measured manually, on a cell-by-cell basis, from the outline of cells stained for α-actinin, a cardiac cytoskeletal protein (Fig. 5E). These geometric measurements were in excellent agreement with the ratiometric SRB approach. A second type of validation experiment measured markers of hypertrophy at message level in lysates (Fig. 5F). The results were in broad agreement with the SRB method, although the relationship was non-linear, as would be expected from the nature of transcript-based markers.

### 3.5 Measuring agonist responses in cardiac fibroblasts

Around half of the cells in the heart are non-myocytes, therefore some degree of fibroblast growth in cultured NRVM monolayers is inevitable. Our isolation protocol is able to reduce the fibroblast population to ~10% under control conditions (Fig. 2A/Fig. 4B), which means that the majority of SRB fluorescence collected from a monolayer will be myocyte-derived. However, a strong pro-growth response of fibroblasts to a test-treatment (e.g. agonist) would skew the monolayer-averaged SRB index away from reporting growth in myocytes. Such an artefactual influence of fibroblasts would become problematic if fibroblasts emitted disproportionally higher SRB fluorescence compared to myocytes, or if their pro-growth responses gauged by SRB ratio were dramatically greater than the monolayer-averaged SRB ratio response.

**Fig. 6:**
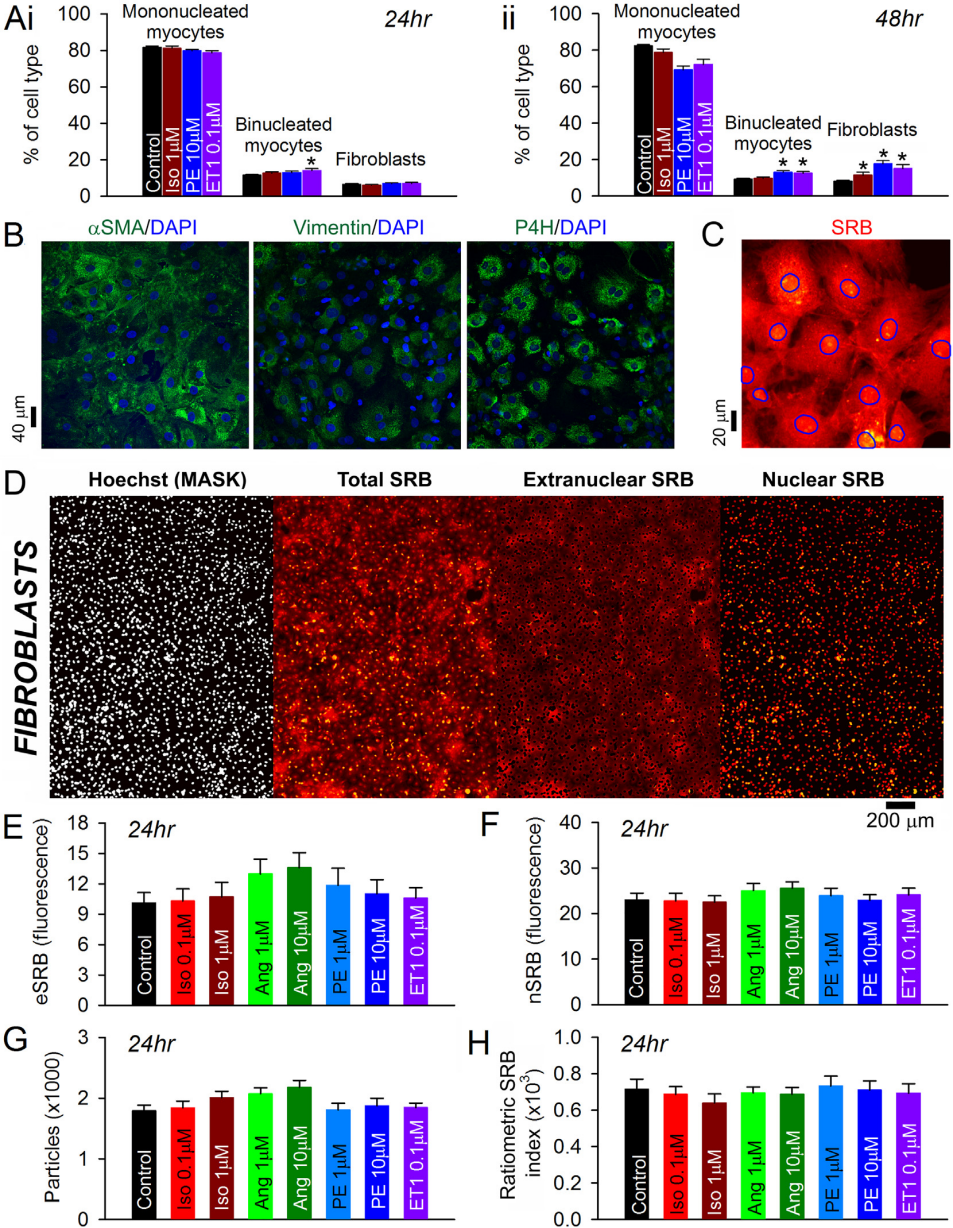
Performing the SRB assay on cardiac fibroblasts. (A) Effect of (i) 24 h and (ii) 48 h treatment with agonists on the population of mono- and binucleated myocytes and fibroblasts. (B) Immunofluorescence confocal image of cells collected at the ‘preplating step’. Staining for markers of fibroblasts or fibroblast-like cells: α smooth muscle actin (αSMA), vimentin and prolyl 4-hydroxylase (P4H). (C) Fibroblasts stained with SRB (×40 magnification). Nuclei outline identified by Hoechst. (D) Imaging of fibroblasts using the automated plate-reader. Hoechst staining yields a binary mask for separating the SRB signal into nuclear and extra-nuclear components (nSRB, eSRB). (E) Effect of chemical agonists on eSRB and (F) nSRB fluorescence following 24 h treatment (n = 16/4). (G) Particle count. (H) Ratiometric SRB index showing lack of effect of agonists on fibroblast growth. Experiments performed on cells obtained from four isolations of 10–24 rat pups, with four technical repeats each. Mean ± SEM.

To test if agonist-evoked changes reported by the SRB index (e.g. Fig. 5D) were likely affected by a substantial expansion of the fibroblast population, nuclear staining was analyzed for cell type distribution (see Fig. 2C/D). Treatment with Iso, PE or ET1 for 24 h did not greatly affect the distribution of cells among subtypes classified as mononucleated myocytes, binucleated myocytes and fibroblasts (Fig. 6Ai). Longer treatment (48 h) expanded the fibroblast sub-population, but these cells remained a minority and unlikely to meaningfully skew the monolayer-derived SRB index (Fig. 6Aii).

To investigate further the degree of SRB binding to fibroblasts, cells collected at the ‘preplating step’ were seeded at low density and cultured for a week. Owing to continued proliferative capacity, this produces cultures greatly enriched in fibroblasts. Indeed, the majority of cells grown in this way stained positively for α smooth muscle actin (αSMA), vimentin and prolyl 4-hydroxylase, which collectively indicate a fibroblast cell type [41] (Fig. 6B). The SRB assay was performed on fibroblasts, with a modification to increase either the gain or exposure time for Hoechst fluorescence acquisition to compensate for weaker nuclear staining (Fig. 6C). To obtain good SRB signal, laser power was increased 3-fold, compared to the settings optimised for ‘myocytes’; this correction indicates that SRB binding to fibroblasts is weaker than to NRVMs. Consequently, the total SRB signal collected from monolayers in experiments such as those shown in Fig. 2, Fig. 3, Fig. 4 is likely to be biased even further towards the myocyte population. To analyze the fibroblast responses to agonists, cells were seeded in 96-well plates and stimulated with Iso, Ang, PE, or ET1 for 24 h. Cells were then stained and imaged using the plate-reader platform (Fig. 6D), and analyzed in terms of eSRB, nSRB and number of nuclei (Fig. 6E–H). At baseline, the ratiometric index was higher in fibroblasts compared to NRVMs, which likely relates to differences in subcellular protein distribution and cell size. None of the agonists tested produced a hypertrophic response in fibroblasts (Fig. 6H). PE and ET1, which produced robust hypertrophy in myocytes, evoked no significant fibroblast growth. Taken together, these findings demonstrate that the bulk of hypertrophic growth described in Figs. 3–5 is attributable to myocytes.

### 3.6 Applying the SRB method to characterize agonist-evoked hypertrophic responses

**Fig. 7:**
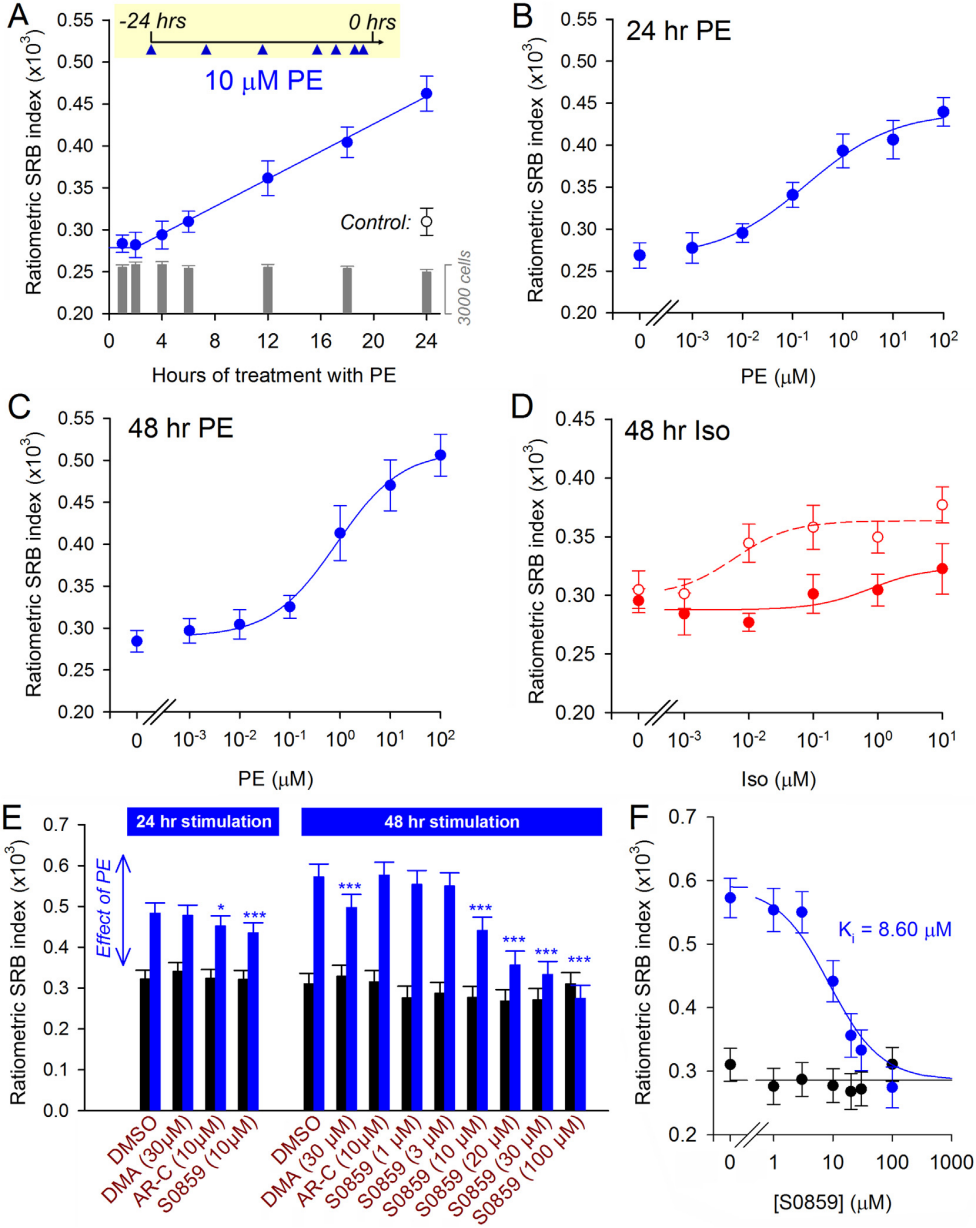
Applications of the ratiometric SRB assay. (A) Time course of hypertrophic response to 10 μM PE applied for the final 1–24 h of incubation (in 100 μM ascorbate). Right axis: myocytes per field of view. Control had no PE added (n = 16/4). (B) Cell growth triggered by 24 h or (C) 48 h treatment with PE (n = 16/4). Best-fit to R_0_ + R_hyp_ × ([PE]^n^)/(EC_50_^n^ + [PE]^n^); R_0_ is baseline, R_hyp_ is maximal increase, n is cooperativity, EC_50_ is the concentration at half-maximal effect. 24 h treatment: 0.266, 0.174, 0.50, 0.185 μM; 48 h treatment: 0.289, 0.223, 0.67, 0.844 μM. (D) Growth triggered by 48 h treatment with Iso; repeated in the presence of calyculin-A (5 nM) (n = 16/4). (E) Screen for anti-hypertrophic effects of drugs (inhibitors of pH_i_-regulating proteins) on 10 μM PE-induced cell growth (n = 25/5). DMA: 5-(*N*,*N*-dimethyl)amiloride, AR-C: AR-C155858. (F) Concentration dependence of anti-hypertrophic effect of S0859 on NRVMs stimulated for 48 h with 10 μM PE. Experiments performed on cells obtained from 5 isolations of 10–24 rat pups, with four technical repeats each. Mean ± SEM.

Measurements of cell size or of specific hypertrophy markers rarely have the resolving power to track time courses of hypertrophic responses with adequate temporal detail, or to generate concentration-response curves which provide essential quantitative pharmacological information about cellular responses. To illustrate the strengths of the SRB method, the time course of the hypertrophic response to 10 μM PE was measured over a 24-hour period. PE (chemically stabilized with 100 μM ascorbate) was added for a final period of incubation ranging from 1 to 24 h. Significant growth was detectable after ~4 h of PE stimulus, beyond which it progressed linearly (Fig. 7A), without a change in cell number.

To determine if the SRB assay has the power to generate concentration-response curves, NRVMs were exposed to a range of concentrations of PE for either 24 or 48 h (Fig. 7B). The method was able to construct concentration-response curves and derive parameters such as EC_50_ (the agonist dose that evokes a half-maximal effect). Moreover, the results highlight a marked difference in the PE response profile probed after 24 versus 48 h of treatment. After the first 24-h period, the PE response could be described with an EC_50_ of 0.2 μM and maximal growth approaching 166% of control. Over the subsequent 24-h period, the apparent EC_50_ shifted to 0.8 μM and maximal growth was inflated to 177% (Fig. 7C). This time-dependent change was not due to a gradual degradation of agonist, because replacing media with freshly prepared PE at the midpoint of 48-hr stimulation did not affect the dose-dependence probed at the end of incubation (Fig. S1). As a plausible model, this time-dependent change could be described by the sum of a fast- and slow-onset process, characterized by a high and low affinity, respectively.

The SRB method was tested for its ability to resolve interactions between drugs. Results shown in Fig. 5D indicated that Iso, alone, was unable to evoke a hypertrophic response. A concentration-response curve obtained over a wider range of drug doses confirmed the lack of any significant effect (Fig. 7D). However, certain modalities of cAMP signaling have been shown to evoke hypertrophy [13], and to expose this effect, the concentration-response curve for Iso was mapped in the presence of the phosphatase PP2A/PP1 inhibitor calyculin-A to enhance the actions of Iso-evoked cAMP signals at the level of phosphoproteins [42]. The synergy between Iso and calyculin-A (5 nM) was manifested after 48 h of treatment (Fig. 7D).

### 3.7 Applying the SRB method to screen for anti-hypertrophic agents

It is well-established that Ca^2+^-driven processes, such as contraction, are subservient to the powerful modulatory effects of pH [43]. The influence of acid-base homeostasis on hypertrophic growth is, however, less well characterized, not least because of limitations of existing techniques to follow pH-related responses over the necessarily longer time line of hypertrophy. Myocyte pH is tightly regulated by transporters, such as Na^+^/H^+^ exchangers (NHE [44]) and Na^+^–HCO_3_^–^ co-transporters (NBC [44]), and their activity can influence Ca^2+^-dependent pathways dually, via direct H^+^ ion signaling as well as through changes in [Na^+^] [27,43,45].

To investigate how dysregulated pH may affect agonist-evoked hypertrophic responses, the SRB assay was performed on NRVMs treated with a panel of inhibitors. For the final 48 h of culture before SRB measurements, NRVMs were treated with one of the following: the NHE1 inhibitor 5-(*N*,*N*-dimethyl)-amiloride (DMA) [46], the NBC inhibitor S0859 [47], the H^+^-monocarboxylate transport (MCT1) inhibitor AR-C155858, or vehicle (DMSO) as control. Hypertrophy was induced by PE (10 μM) added either at the start of this 48 h incubation period, or for the final 24 h. Thus, this protocol interrogated the effect of inhibitors of pH regulation on the growth response to a 24 h or 48 h PE stimulus. Inhibition of MCT1 had no substantial effect on growth, which may be expected from the predominantly oxidative nature of myocyte metabolism. Inhibition of NHE1 modestly ablated PE-evoked growth after longer (48 h) stimulation only. S0859 at 10 μM was found to modestly reduce the extent of hypertrophy evoked by 24 h stimulation with PE, and this anti-hypertrophic effect became very pronounced with 48 h PE stimulation (Fig. 7E; Table S2). The inhibitory effect followed a concentration-dependence described by an inhibitory constant (K_i_) of 9 μM (Fig. 7F), a dose that is expected to produce 85% inhibition of NBC activity [47]. Thus, we have demonstrated the merit of the SRB-based method in screening a panel of drugs for hitherto uncharacterized anti-hypertrophic effects.

## 4 Discussion

We present a method for quantifying cellular hypertrophy by means of a ratiometric, fluorescence-based assay that yields high-throughput data with good resolving power over a wide dynamic range, and can be applied to studies of cardiomyocyte growth *in vitro*. Using the method, we were able to provide a more complete pharmacological description of agonist-induced hypertrophy, characterize the effect of cell density on growth, and identify a novel anti-hypertrophic agent, S0859, as proof-of-principle compatibility with screening pipelines.

Assays based on *fluorescence* are generally more accurate and sensitive than those measuring absorbance. The source of fluorescence is protein-bound SRB, providing a linear measure of biomass, irrespective of the type of protein (Fig. 1). Build-up of cellular protein reliably tracks the progression of agonist-induced hypertrophy with good temporal resolution (e.g. Fig. 7A), in contrast to the often complex time course and non-linearity of specific markers. By collecting total SRB fluorescence, it is possible to assess overall growth in all dimensions, and not just across the horizontal plane, as would normally be done with geometric measures of cell area. The SRB spectrum does not overlap with cellular auto-fluorescence or UV-excitable nuclear dyes, allowing accurate resolution with minimal background. Moreover, the red-shifted peak of SRB fluorescence is compatible for use with cells expressing fluorescent proteins, such as GFP or CFP.

As a modification to other SRB-based methods, our protocol measures SRB bound to proteins in situ in order to preserve information relating to its subcellular distribution (Fig. 1, Fig. 2E, Fig. 3A, Fig. 4A, Fig. 6D). This enables image analysis algorithms to segregate the signal into its extra-nuclear and nuclear components for *ratiometric* analysis (Fig. 2E). The rationale for this approach is that the nuclear signal, averaged for the field-of-view, is independent of cell number and insensitive to cell size (Fig. 1C) or growth-stimulating agents (Figs. 5B and 6F), whereas the extra-nuclear signal, which includes areas covered by cells and their background, provides a robust readout of protein biomass. The ratio of these signals eliminates extraneous variables, such as those related to the imaging equipment, because such factors would affect nuclear and extra-nuclear fluorescence equally. Normalizing this ratio to the number of cells provides a measure of cellular growth. We have adapted the image analysis to characterize the geometry of particles identified by nuclear staining (Fig. 2C) and use this to estimate the number of fibroblasts, binucleated myocytes and mononucleated myocytes. Information about the distribution of cells by cell type is relevant for performing quality-control of isolations and for monitoring the responses of different sub-populations to experimental interventions. In principle, the SRB assay could be adapted to include a step for staining fibroblasts and obtain a readout of myocyte-only growth by subtraction. Critically, immunofluorescence would add a complex step to the protocol, but its benefit depends on the quality of markers. Antibodies against certain (nominally) fibroblast markers, such as vimentin [43], also produce a degree of myocyte staining [44,45]. Other markers, such as those used herein (Fig. 6B) may not identify all fibroblast-like cells, thus necessitating the use of a combination of markers which would exacerbate the protocol complexity and may use-up the available spectrum for SRB measurements. The suitability of fibroblast markers also relies on their ability to generate a binary mask for image subtraction. Some markers, such as P4H (Fig. 6B), produce punctate staining, which does not cover the entire fibroblast volume, making masking impractical. Our isolation protocol has been optimized to yield a low fibroblast count (10%), and this was not dramatically altered by pro-hypertrophic treatments (Fig. 6A) or cell density (Fig. 4B). Considering (i) the low fibroblast contamination, (ii) the observation that fibroblast-derived SRB fluorescence is weaker than the myocyte-derived signal, and (iii) the lack of a pro-hypertrophic response of fibroblasts to a panel of agonists, the monolayer-wide SRB index can adequately describe myocyte growth without necessitating further corrections. This approximation is justified in light of the nature of high-throughput screens, which normally require protocols to be robust, rapid and not onerous.

A truly ratiometric index is expected to yield similar values under comparable conditions, even when acquired using different measurement devices. Supporting this, the responses of NRVMs to a particular chemical stimulus were comparable between imaging platforms (Figs. 3G and 5D). Moreover, under control conditions at average cell density, the ratiometric SRB index of growth was ~0.3 × 10^3^ on two different imaging systems, suggesting that results of this assay could be compared between different laboratories. This ratiometric index can also serve as a quality-control of cell isolations, on the basis that stresses accumulating during poorly executed isolations will become apparent from the size of myocytes (e.g. seeding density, Fig. 4C).

Imaging large fields-of-view generates data with excellent *resolving power* over a wide *dynamic range*. These properties are necessary for determining concentration-response curves, response time courses or interactions between drugs (Fig. 7A–D). Thus, it is possible to characterize hypertrophic responses to a finer level of detail. We were able to show that the hypertrophic response to PE begins within 4 h of treatment, and then progresses linearly for the first 24 h (Fig. 7A). Myocytes continue to grow over the subsequent 24-hour period, but the PE response was characterized by an apparent shift towards a higher EC_50_ (Fig. 7B–C), possibly indicating the activation of a delayed, low-affinity mechanism. In contrast to these more complex effects of PE, a different IP_3_-mobilising agonist ET1 produced a peak hypertrophic response within 24 h (Fig. 5). These subtle differences in agonist responses may provide new insights into the signaling mechanisms that underpin disease-related hypertrophy, and identify better ways of targeting these therapeutically.

The simple workflow for staining and imaging, combined with the computationally-inexpensive offline analysis, are compatible with up-scaling the method for use in *high-throughput* studies with automated plate readers (e.g. Figs. 4–7). Thus, libraries of drugs can be screened for their pro- or anti-hypertrophic actions. Compared to manually-generated geometrical measures of cell size, the present method avoids potential errors of user-bias and generates data considerably faster. The computationally-minimal workflow is a considerable advantage over computationally-intensive segmentation programs for automated cell size measurements [[16], [17], [18]]. To illustrate the utility of the method, we used the assay to screen drugs for anti-hypertrophic actions. The proof-of-principle experiment was performed on a panel of substances that are canonical inhibitors of pH-regulating proteins. Our rationale for choosing this class of drugs was that intracellular pH is a well-established modulator of Ca^2+^-dependent cardiac functions, such as excitation-contraction coupling [46], and could, in principle, also influence agonist-induced hypertrophy. PE-induced hypertrophy was curtailed dose-dependently by S0859, an inhibitor of Na^+^–HCO_3_^–^ transport (NBC) first characterized in the heart by our laboratory [47]. A previous study [48] has demonstrated an off-target inhibitory effect of S0859 on MCT, but this is unlikely to underlie its anti-hypertrophic effect because the potent and selective MCT1 inhibitor, AR-C155858, did not significantly attenuate the pro-growth effect of PE. NBC actively fine-tunes pH in the myocyte's dyadic spaces [49], and is also a major Na^+^-entry pathway into the cell [50]. Consequently, the target of S0859 is in prime location to influence Ca^2+^ signals via actions on intracellular pH and [Na^+^]. Although others have implicated NHE1 in various models of hypertrophy [51] and described its pharmacological blockers as being anti-hypertrophic [[52], [53], [54]], the NHE1 inhibitor DMA produced only a modest anti-hypertrophic effect that was significant only with longer PE stimulation. Compared to NBC, NHE1 produces larger corrective H^+^-fluxes at low intracellular pH, but activity near resting pH is comparable between the two transporters. Since NHE is more remote from dyadic spaces than NBC [49], it is possible that the target of S0859 is more privileged in influencing agonist-evoked pro-hypertrophic Ca^2+^ cascades.

On the basis of our results, the SRB method is confirmed to have good *statistical power* to resolve differences. A widely recognized issue that affects the reproducibility of data is pseudo-replication [28,55], which occurs when there is substantial variation in the readouts obtained between different batches of cells, yet technical repeats are treated as independent experiments. Data that are poorly calibratable and strongly influenced by extraneous (e.g. equipment-related) variables tend to ‘cluster’ within each biological repeat (i.e. high intra-class correlation, ICC) [28] and are, therefore, more prone to producing false-positive inferences. Ratiometric methods, in general, reduce ICC and thus increase the resolving power of statistical analyses that incorporate hierarchical methodologies to account for clustering. This is demonstrated in the statistical analyses presented in Tables S1–S2. For instance, the improved resolving power attained with the SRB assay is manifested by its ability to resolve an interaction between β agonists and phosphatase inhibitors (Fig. 7D).

In conclusion, our simple method, readily implemented as an end-point in experimental protocols, can robustly quantify pro- or anti-hypertrophic effects of pharmacological or genetic interventions.

## Funding

British Heart Foundation Programme Grant (to PS) [grant number RG/15/9/31534].

## Conflicts of interest

None to declare.

## Appendix A Supplementary data

Supplementary material Image 1

## References

[1] Nakamura M., Sadoshima J. (2018). Mechanisms of physiological and pathological cardiac hypertrophy. Nat. Rev. Cardiol..

[2] Levy D., Garrison R.J., Savage D.D., Kannel W.B., Castelli W.P. (1990). Prognostic implications of echocardiographically determined left ventricular mass in the Framingham Heart Study. N. Engl. J. Med..

[3] Lorell B.H., Carabello B.A. (2000). Left ventricular hypertrophy: pathogenesis, detection, and prognosis. Circulation.

[4] Akazawa H., Komuro I. (2003). Roles of cardiac transcription factors in cardiac hypertrophy. Circ. Res..

[5] Sugden P.H. (1999). Signaling in myocardial hypertrophy: life after calcineurin?. Circ. Res..

[6] van Berlo J.H., Maillet M., Molkentin J.D. (2013). Signaling effectors underlying pathologic growth and remodeling of the heart. J. Clin. Invest..

[7] Taglieri D.M., Monasky M.M., Knezevic I., Sheehan K.A., Lei M., Wang X., Chernoff J., Wolska B.M., Ke Y., Solaro R.J. (2011). Ablation of p21-activated kinase-1 in mice promotes isoproterenol-induced cardiac hypertrophy in association with activation of Erk1/2 and inhibition of protein phosphatase 2A. J. Mol. Cell. Cardiol..

[8] Saadane N., Alpert L., Chalifour L.E. (1999). Expression of immediate early genes, GATA-4, and Nkx-2.5 in adrenergic-induced cardiac hypertrophy and during regression in adult mice. Br. J. Pharmacol..

[9] Deng K.Q., Wang A., Ji Y.X., Zhang X.J., Fang J., Zhang Y., Zhang P., Jiang X., Gao L., Zhu X.Y., Zhao Y., Gao L., Yang Q., Zhu X.H., Wei X., Pu J., Li H. (2016). Suppressor of IKKvarepsilon is an essential negative regulator of pathological cardiac hypertrophy. Nat. Commun..

[10] Simpson P., McGrath A., Savion S. (1982). Myocyte hypertrophy in neonatal rat heart cultures and its regulation by serum and by catecholamines. Circ. Res..

[11] Simpson P. (1985). Stimulation of hypertrophy of cultured neonatal rat heart cells through an alpha 1-adrenergic receptor and induction of beating through an alpha 1- and beta 1-adrenergic receptor interaction. Evidence for independent regulation of growth and beating. Circ. Res..

[12] Glembotski C.C. (2013). Classic studies of cultured cardiac myocyte hypertrophy: interview with a transformer. Circ. Res..

[13] Zoccarato A., Surdo N.C., Aronsen J.M., Fields L.A., Mancuso L., Dodoni G., Stangherlin A., Livie C., Jiang H., Sin Y.Y., Gesellchen F., Terrin A., Baillie G.S., Nicklin S.A., Graham D., Szabo-Fresnais N., Krall J., Vandeput F., Movsesian M., Furlan L., Corsetti V., Hamilton G., Lefkimmiatis K., Sjaastad I., Zaccolo M. (2015). Cardiac hypertrophy is inhibited by a local pool of cAMP regulated by phosphodiesterase 2. Circ. Res..

[14] Volkers M., Toko H., Doroudgar S., Din S., Quijada P., Joyo A.Y., Ornelas L., Joyo E., Thuerauf D.J., Konstandin M.H., Gude N., Glembotski C.C., Sussman M.A. (2013). Pathological hypertrophy amelioration by PRAS40-mediated inhibition of mTORC1. Proc. Natl. Acad. Sci. U. S. A..

[15] Kehat I., Molkentin J.D. (2010). Molecular pathways underlying cardiac remodeling during pathophysiological stimulation. Circulation.

[16] Bass G.T., Ryall K.A., Katikapalli A., Taylor B.E., Dang S.T., Acton S.T., Saucerman J.J. (2012). Automated image analysis identifies signaling pathways regulating distinct signatures of cardiac myocyte hypertrophy. J. Mol. Cell. Cardiol..

[17] Ryall K.A., Saucerman J.J. (2015). Automated microscopy of cardiac myocyte hypertrophy: a case study on the role of intracellular alpha-adrenergic receptors. Methods Mol. Biol..

[18] Reid B.G., Stratton M.S., Bowers S., Cavasin M.A., Demos-Davies K.M., Susano I., McKinsey T.A. (2016). Discovery of novel small molecule inhibitors of cardiac hypertrophy using high throughput, high content imaging. J. Mol. Cell. Cardiol..

[19] Cao B., Yu Q., Zhao W., Tang Z., Cong B., Du J., Lu J., Zhu X., Ni X. (2016). Kallikrein-related peptidase 8 is expressed in myocardium and induces cardiac hypertrophy. Sci. Rep..

[20] Chien K.R., Knowlton K.U., Zhu H., Chien S. (1991). Regulation of cardiac gene expression during myocardial growth and hypertrophy: molecular studies of an adaptive physiologic response. FASEB J..

[21] Harvey P.A., Leinwand L.A. (2011). The cell biology of disease: cellular mechanisms of cardiomyopathy. J. Cell Biol..

[22] Gardner D.G. (2003). Natriuretic peptides: markers or modulators of cardiac hypertrophy?. Trends Endocrinol. Metab..

[23] Zimmer H.G., Steinkopff G., Gerlach E. (1972). Changes of protein synthesis in the hypertrophying rat heart. Pflugers Arch..

[24] Tirziu D., Chorianopoulos E., Moodie K.L., Palac R.T., Zhuang Z.W., Tjwa M., Roncal C., Eriksson U., Fu Q., Elfenbein A., Hall A.E., Carmeliet P., Moons L., Simons M. (2007). Myocardial hypertrophy in the absence of external stimuli is induced by angiogenesis in mice. J. Clin. Invest..

[25] Branco A.F., Pereira S.P., Gonzalez S., Gusev O., Rizvanov A.A., Oliveira P.J. (2015). Gene expression profiling of H9c2 myoblast differentiation towards a cardiac-like phenotype. PLoS ONE.

[26] Vichai V., Kirtikara K. (2006). Sulforhodamine B colorimetric assay for cytotoxicity screening. Nat. Protoc..

[27] Ford K.L., Moorhouse E.L., Bortolozzi M., Richards M.A., Swietach P., Vaughan-Jones R.D. (2017). Regional acidosis locally inhibits but remotely stimulates Ca2+ waves in ventricular myocytes. Cardiovasc. Res..

[28] Sikkel M.B., Francis D.P., Howard J., Gordon F., Rowlands C., Peters N.S., Lyon A.R., Harding S.E., MacLeod K.T. (2017). Hierarchical statistical techniques are necessary to draw reliable conclusions from analysis of isolated cardiomyocyte studies. Cardiovasc. Res..

[29] Skehan P., Storeng R., Scudiero D., Monks A., McMahon J., Vistica D., Warren J.T., Bokesch H., Kenney S., Boyd M.R. (1990). New colorimetric cytotoxicity assay for anticancer-drug screening. J. Natl. Cancer Inst..

[30] Monks A., Scudiero D., Skehan P., Shoemaker R., Paull K., Vistica D., Hose C., Langley J., Cronise P., Vaigro-Wolff A. (1991). Feasibility of a high-flux anticancer drug screen using a diverse panel of cultured human tumor cell lines. J. Natl. Cancer Inst..

[31] Han F., Fan L., Wang X., Li W. (2012). Sulforhodamine B restaining as a whole-cell label allows visualizing one more fluorochrome and its application in assaying protein nucleocytoplasmic distribution. Cytometry A.

[32] Grynkiewicz G., Poenie M., Tsien R.Y. (1985). A new generation of Ca2+ indicators with greatly improved fluorescence properties. J. Biol. Chem..

[33] Korecky B., Sweet S., Rakusan K. (1979). Number of nuclei in mammalian cardiac myocytes. Can. J. Physiol. Pharmacol..

[34] Zobel C., Kassiri Z., Nguyen T.T., Meng Y., Backx P.H. (2002). Prevention of hypertrophy by overexpression of Kv4.2 in cultured neonatal cardiomyocytes. Circulation.

[35] Menaouar A., Florian M., Wang D., Danalache B., Jankowski M., Gutkowska J. (2014). Anti-hypertrophic effects of oxytocin in rat ventricular myocytes. Int. J. Cardiol..

[36] El-Armouche A., Eschenhagen T. (2009). Beta-adrenergic stimulation and myocardial function in the failing heart. Heart Fail. Rev..

[37] Stewart A.F., Rokosh D.G., Bailey B.A., Karns L.R., Chang K.C., Long C.S., Kariya K., Simpson P.C. (1994). Cloning of the rat alpha 1C-adrenergic receptor from cardiac myocytes. Alpha 1C, alpha 1B, and alpha 1D mRNAs are present in cardiac myocytes but not in cardiac fibroblasts. Circ. Res..

[38] Zhang L., Malik S., Pang J., Wang H., Park K.M., Yule D.I., Blaxall B.C., Smrcka A.V. (2013). Phospholipase cepsilon hydrolyzes perinuclear phosphatidylinositol 4-phosphate to regulate cardiac hypertrophy. Cell.

[39] Gray M.O., Long C.S., Kalinyak J.E., Li H.T., Karliner J.S. (1998). Angiotensin II stimulates cardiac myocyte hypertrophy via paracrine release of TGF-beta 1 and endothelin-1 from fibroblasts. Cardiovasc. Res..

[40] Sugden P.H., Fuller S.J., Weiss S.C., Clerk A. (2008). Glycogen synthase kinase 3 (GSK3) in the heart: a point of integration in hypertrophic signalling and a therapeutic target? A critical analysis. Br. J. Pharmacol..

[41] Ivey M.J., Tallquist M.D. (2016). Defining the cardiac fibroblast. Circ. J..

[42] Burdyga A., Surdo N.C., Monterisi S., Di Benedetto G., Grisan F., Penna E., Pellegrini L., Zaccolo M., Bortolozzi M., Swietach P., Pozzan T., Lefkimmiatis K. (2018). Phosphatases control PKA-dependent functional microdomains at the outer mitochondrial membrane. Proc. Natl. Acad. Sci. U. S. A..

[43] Tarbit E., Singh I., Peart J.N., Rose'Meyer R.B. (2019). Biomarkers for the identification of cardiac fibroblast and myofibroblast cells. Heart Fail. Rev..

[44] Osinska H.E., Lemanski L.F. (1989). Immunofluorescent localization of desmin and vimentin in developing cardiac muscle of Syrian hamster. Anat. Rec..

[45] LaFramboise W.A., Scalise D., Stoodley P., Graner S.R., Guthrie R.D., Magovern J.A., Becich M.J. (2007). Cardiac fibroblasts influence cardiomyocyte phenotype in vitro. Am. J. Phys. Cell Physiol..

[46] Vaughan-Jones R.D., Spitzer K.W., Swietach P. (2009). Intracellular pH regulation in heart. J. Mol. Cell. Cardiol..

[47] Ch'en F.F., Villafuerte F.C., Swietach P., Cobden P.M., Vaughan-Jones R.D. (2008). S0859, an N-cyanosulphonamide inhibitor of sodium-bicarbonate cotransport in the heart. Br. J. Pharmacol..

[48] Heidtmann H., Ruminot I., Becker H.M., Deitmer J.W. (2015). Inhibition of monocarboxylate transporter by N-cyanosulphonamide S0859. Eur. J. Pharmacol..

[49] Garciarena C.D., Ma Y.L., Swietach P., Huc L., Vaughan-Jones R.D. (2013). Sarcolemmal localisation of Na+/H+ exchange and Na+-HCO3- co-transport influences the spatial regulation of intracellular pH in rat ventricular myocytes. J. Physiol..

[50] Garciarena C.D., Youm J.B., Swietach P., Vaughan-Jones R.D. (2013). H(+)-activated Na(+) influx in the ventricular myocyte couples ca(2)(+)-signalling to intracellular pH. J. Mol. Cell. Cardiol..

[51] Cingolani H.E., Ennis I.L. (2007). Sodium-hydrogen exchanger, cardiac overload, and myocardial hypertrophy. Circulation.

[52] Baartscheer A., Schumacher C.A., van Borren M.M., Belterman C.N., Coronel R., Opthof T., Fiolet J.W. (2005). Chronic inhibition of Na+/H+-exchanger attenuates cardiac hypertrophy and prevents cellular remodeling in heart failure. Cardiovasc. Res..

[53] Chen L., Chen C.X., Gan X.T., Beier N., Scholz W., Karmazyn M. (2004). Inhibition and reversal of myocardial infarction-induced hypertrophy and heart failure by NHE-1 inhibition. Am. J. Physiol. Heart Circ. Physiol..

[54] Yoshida H., Karmazyn M. (2000). Na(+)/H(+) exchange inhibition attenuates hypertrophy and heart failure in 1-wk postinfarction rat myocardium. Am. J. Physiol. Heart Circ. Physiol..

[55] Lazic S.E. (2010). The problem of pseudoreplication in neuroscientific studies: is it affecting your analysis?. BMC Neurosci..

