# Supplementary material for: A high-throughput ratiometric method for imaging hypertrophic growth in cultured primary cardiac myocytes
Source: J Mol Cell Cardiol. 2019 May;130:184–96. doi: 10.1016/j.yjmcc.2019.04.001 (PMC6520438; doi:10.1016/j.yjmcc.2019.04.001)
Supplement: Supplementary file 1 — Supplementary material [file mmc1.docx]

**A HIGH-THROUGHPUT RATIOMETRIC METHOD FOR IMAGING HYPERTROPHIC GROWTH
IN CULTURED PRIMARY CARDIAC MYOCYTES**

**SUPPLEMENT**

*Contents:*

- Supplementary Table S1 and S2
- Supplementary Methods
- Supplementary Figure S1

**Supplementary Table S1:** One-way ANOVA analysis of Fig 5 using hierarchical methodology

| **Treatment** | **Comparison** | **Variable** | **Estimate** | **Std. Error** | **df** | **t value** | **lower** | **upper** | **Pr(>\|t\|)** | **Bonferroni p-value** | |
| --- | --- | --- | --- | --- | --- | --- | --- | --- | --- | --- | --- |
| 24 hours | vs Iso 0.1uM | eSRB | -687.004 | 632.3393 | 110.0844 | -1.08645 | -1940.14 | 566.1332 | 0.279654 | 1 | N/S |
| 24 hours | vs Iso 1uM | eSRB | -839.932 | 602.1863 | 110.0106 | -1.3948 | -2033.32 | 353.4582 | 0.165885 | 1 | N/S |
| 24 hours | vs Ang 1uM | eSRB | -1217.4 | 602.1863 | 110.0106 | -2.02163 | -2410.79 | -24.0082 | 0.045642 | 1 | N/S |
| 24 hours | vs Ang 10uM | eSRB | -1446.01 | 602.1863 | 110.0106 | -2.40127 | -2639.4 | -252.623 | 0.018015 | 0.518828 | N/S |
| **24 hours** | **vs PE 1uM** | **eSRB** | **-5444.89** | **602.1863** | **110.0106** | **-9.04187** | **-6638.28** | **-4251.5** | **6.1E-15** | **1.76E-13** | ******* |
| **24 hours** | **vs PE 10uM** | **eSRB** | **-6761.98** | **602.1863** | **110.0106** | **-11.2291** | **-7955.38** | **-5568.59** | **5.88E-20** | **1.69E-18** | ******* |
| **24 hours** | **vs ET1 10nM** | **eSRB** | **-5609.07** | **790.1606** | **107.2065** | **-7.09864** | **-7175.43** | **-4042.7** | **1.43E-10** | **4.13E-09** | ******* |
| **24 hours** | **vs ET1 100nM** | **eSRB** | **-5391.62** | **584.3196** | **116.9998** | **-9.22717** | **-6548.83** | **-4234.4** | **1.44E-15** | **4.14E-14** | ******* |
| 24 hours | vs Iso 0.1uM | nSRB | -3199.87 | 1104.241 | 110.2776 | -2.8978 | -5388.16 | -1011.59 | 0.004534 | 0.13058 | N/S |
| 24 hours | vs Iso 1uM | nSRB | -2811.48 | 1051.528 | 110.2355 | -2.67371 | -4895.31 | -727.647 | 0.008642 | 0.248897 | N/S |
| 24 hours | vs Ang 1uM | nSRB | -3685.99 | 1051.528 | 110.2355 | -3.50537 | -5769.82 | -1602.16 | 0.00066 | 0.01902 | * |
| 24 hours | vs Ang 10uM | nSRB | -4162.58 | 1051.528 | 110.2355 | -3.9586 | -6246.41 | -2078.75 | 0.000134 | 0.003855 | ** |
| 24 hours | vs PE 1uM | nSRB | -1064.24 | 1051.528 | 110.2355 | -1.01209 | -3148.07 | 1019.59 | 0.313711 | 1 | N/S |
| 24 hours | vs PE 10uM | nSRB | -2742.23 | 1051.528 | 110.2355 | -2.60785 | -4826.06 | -658.396 | 0.010373 | 0.298752 | N/S |
| 24 hours | vs ET1 10nM | nSRB | -3455.19 | 1411.714 | 115.4097 | -2.44751 | -6251.42 | -658.961 | 0.015891 | 0.45766 | N/S |
| 24 hours | vs ET1 100nM | nSRB | -3139.3 | 1031.199 | 116.3171 | -3.04432 | -5181.67 | -1096.94 | 0.002884 | 0.083071 | N/S |
| 24 hours | vs Iso 0.1uM | Cells | -19.9897 | 150.8883 | 110.0197 | -0.13248 | -319.014 | 279.0349 | 0.894847 | 1 | N/S |
| 24 hours | vs Iso 1uM | Cells | -136.045 | 143.7136 | 109.8595 | -0.94664 | -420.856 | 148.7656 | 0.3459 | 1 | N/S |
| 24 hours | vs Ang 1uM | Cells | -217.128 | 143.7136 | 109.8595 | -1.51084 | -501.939 | 67.68231 | 0.1337 | 1 | N/S |
| 24 hours | vs Ang 10uM | Cells | -222.045 | 143.7136 | 109.8595 | -1.54505 | -506.856 | 62.76564 | 0.125209 | 1 | N/S |
| 24 hours | vs PE 1uM | Cells | -366.545 | 143.7136 | 109.8595 | -2.55052 | -651.356 | -81.7344 | 0.012133 | 0.349437 | N/S |
| 24 hours | vs PE 10uM | Cells | -463.462 | 143.7136 | 109.8595 | -3.2249 | -748.272 | -178.651 | 0.00166 | 0.047809 | * |
| 24 hours | vs ET1 10nM | Cells | -212.273 | 179.8009 | 83.81103 | -1.1806 | -569.838 | 145.2925 | 0.241101 | 1 | N/S |
| 24 hours | vs ET1 100nM | Cells | -121.367 | 136.5211 | 115.0963 | -0.889 | -391.787 | 149.0524 | 0.375857 | 1 | N/S |
| 24 hours | vs Iso 0.1uM | SBR ratio | 0.010749 | 0.020898 | 110.4184 | 0.514339 | -0.03066 | 0.052161 | 0.608043 | 1 | N/S |
| 24 hours | vs Iso 1uM | SBR ratio | 0.01109 | 0.019902 | 110.3309 | 0.557214 | -0.02835 | 0.050529 | 0.57851 | 1 | N/S |
| 24 hours | vs Ang 1uM | SBR ratio | 0.016383 | 0.019902 | 110.3309 | 0.823164 | -0.02306 | 0.055822 | 0.41219 | 1 | N/S |
| 24 hours | vs Ang 10uM | SBR ratio | 0.01745 | 0.019902 | 110.3309 | 0.876805 | -0.02199 | 0.05689 | 0.382498 | 1 | N/S |
| **24 hours** | **vs PE 1uM** | **SBR ratio** | **-0.13496** | **0.019902** | **110.3309** | **-6.78143** | **-0.1744** | **-0.09552** | **6.15E-10** | **1.77E-08** | ******* |
| **24 hours** | **vs PE 10uM** | **SBR ratio** | **-0.13992** | **0.019902** | **110.3309** | **-7.03058** | **-0.17936** | **-0.10048** | **1.81E-10** | **5.21E-09** | ******* |
| **24 hours** | **vs ET1 10nM** | **SBR ratio** | **-0.13168** | **0.02581** | **102.2914** | **-5.102** | **-0.18287** | **-0.08049** | **1.55E-06** | **4.48E-05** | ******* |
| **24 hours** | **vs ET1 100nM** | **SBR ratio** | **-0.14656** | **0.019209** | **116.8459** | **-7.62996** | **-0.1846** | **-0.10852** | **6.94E-12** | **2E-10** | ******* |
| 48 hours | vs Iso 0.1uM | eSRB | -1027.93 | 1150.135 | 112.7438 | -0.89375 | -3306.61 | 1250.753 | 0.373362 | 1 | N/S |
| 48 hours | vs Iso 1uM | eSRB | -1080.85 | 1124.323 | 112.7268 | -0.96134 | -3308.4 | 1146.691 | 0.33844 | 1 | N/S |
| 48 hours | vs Ang 1uM | eSRB | -2067.24 | 1124.323 | 112.7268 | -1.83865 | -4294.78 | 160.3047 | 0.068599 | 1 | N/S |
| 48 hours | vs Ang 10uM | eSRB | -1201.44 | 1124.323 | 112.7268 | -1.06859 | -3428.98 | 1026.106 | 0.287538 | 1 | N/S |
| **48 hours** | **vs PE 1uM** | **eSRB** | **-6015.13** | **1124.323** | **112.7268** | **-5.35** | **-8242.68** | **-3787.59** | **4.67E-07** | **1.34E-05** | ******* |
| **48 hours** | **vs PE 10uM** | **eSRB** | **-9841.2** | **1124.323** | **112.7268** | **-8.753** | **-12068.7** | **-7613.66** | **2.35E-14** | **6.78E-13** | ******* |
| **48 hours** | **vs ET1 10nM** | **eSRB** | **-5666.73** | **1442.074** | **93.55326** | **-3.92957** | **-8530.18** | **-2803.28** | **0.000163** | **0.004692** | ******* |
| **48 hours** | **vs ET1 100nM** | **eSRB** | **-6331.6** | **1073.287** | **117.6791** | **-5.89926** | **-8457.06** | **-4206.14** | **3.59E-08** | **1.03E-06** | ******* |
| 48 hours | vs Iso 0.1uM | nSRB | -1657.59 | 1648.509 | 113.0133 | -1.00551 | -4923.58 | 1608.395 | 0.316799 | 1 | N/S |
| 48 hours | vs Iso 1uM | nSRB | -2070.46 | 1611.472 | 113.0096 | -1.28483 | -5263.08 | 1122.149 | 0.20148 | 1 | N/S |
| 48 hours | vs Ang 1uM | nSRB | -3356 | 1611.472 | 113.0096 | -2.08257 | -6548.62 | -163.39 | 0.039547 | 1 | N/S |
| 48 hours | vs Ang 10uM | nSRB | -1947.7 | 1611.472 | 113.0096 | -1.20865 | -5140.32 | 1244.91 | 0.229321 | 1 | N/S |
| 48 hours | vs PE 1uM | nSRB | -1893.81 | 1611.472 | 113.0096 | -1.1752 | -5086.42 | 1298.804 | 0.242383 | 1 | N/S |
| 48 hours | vs PE 10uM | nSRB | -5115.77 | 1611.472 | 113.0096 | -3.17459 | -8308.38 | -1923.15 | 0.001934 | 0.055691 | N/S |
| 48 hours | vs ET1 10nM | nSRB | -1055.98 | 2215.364 | 118.9715 | -0.47666 | -5442.64 | 3330.668 | 0.634476 | 1 | N/S |
| 48 hours | vs ET1 100nM | nSRB | -3074.09 | 1589.144 | 117.5556 | -1.93443 | -6221.15 | 72.97304 | 0.055463 | 1 | N/S |
| 48 hours | vs Iso 0.1uM | Cells | -256.193 | 159.7924 | 108.3846 | -1.60328 | -572.916 | 60.53085 | 0.111782 | 1 | N/S |
| 48 hours | vs Iso 1uM | Cells | -172.5 | 156.2122 | 108.3212 | -1.10427 | -482.129 | 137.1292 | 0.271924 | 1 | N/S |
| 48 hours | vs Ang 1uM | Cells | -364 | 156.2122 | 108.3212 | -2.33016 | -673.629 | -54.3708 | 0.02165 | 0.62353 | N/S |
| 48 hours | vs Ang 10uM | Cells | -384.667 | 156.2122 | 108.3212 | -2.46246 | -694.296 | -75.0375 | 0.015375 | 0.442786 | N/S |
| 48 hours | vs PE 1uM | Cells | -383.75 | 156.2122 | 108.3212 | -2.45659 | -693.379 | -74.1208 | 0.015614 | 0.449686 | N/S |
| 48 hours | vs PE 10uM | Cells | -107.167 | 156.2122 | 108.3212 | -0.68603 | -416.796 | 202.4625 | 0.494158 | 1 | N/S |
| 48 hours | vs ET1 10nM | Cells | -468.999 | 187.5058 | 52.16792 | -2.50125 | -845.228 | -92.7699 | 0.015552 | 0.447901 | N/S |
| 48 hours | vs ET1 100nM | Cells | -120.708 | 144.8818 | 109.6835 | -0.83315 | -407.839 | 166.4231 | 0.406572 | 1 | N/S |
| 48 hours | vs Iso 0.1uM | SBR ratio | 0.008895 | 0.023717 | 110.9967 | 0.375058 | -0.0381 | 0.055893 | 0.708333 | 1 | N/S |
| 48 hours | vs Iso 1uM | SBR ratio | 0.002258 | 0.023185 | 110.9662 | 0.097401 | -0.04369 | 0.048202 | 0.922583 | 1 | N/S |
| 48 hours | vs Ang 1uM | SBR ratio | -0.00469 | 0.023185 | 110.9662 | -0.20212 | -0.05063 | 0.041257 | 0.840195 | 1 | N/S |
| 48 hours | vs Ang 10uM | SBR ratio | 0.011989 | 0.023185 | 110.9662 | 0.517111 | -0.03395 | 0.057933 | 0.606108 | 1 | N/S |
| **48 hours** | **vs PE 1uM** | **SBR ratio** | **-0.14736** | **0.023185** | **110.9662** | **-6.35595** | **-0.19331** | **-0.10142** | **4.72E-09** | **1.36E-07** | ******* |
| **48 hours** | **vs PE 10uM** | **SBR ratio** | **-0.26725** | **0.023185** | **110.9662** | **-11.5269** | **-0.3132** | **-0.22131** | **1.09E-20** | **3.14E-19** | ******* |
| **48 hours** | **vs ET1 10nM** | **SBR ratio** | **-0.13407** | **0.02897** | **75.12766** | **-4.62769** | **-0.19178** | **-0.07636** | **1.51E-05** | **0.000436** | ******* |
| **48 hours** | **vs ET1 100nM** | **SBR ratio** | **-0.17909** | **0.021877** | **115.1066** | **-8.18607** | **-0.22242** | **-0.13575** | **4.11E-13** | **1.18E-11** | ******* |

**Supplementary Table S2:** One-way ANOVA analysis of Fig 7E using hierarchical methodology

| **Stimulus duration** | **Treatment** | **Variable** | **Drug vs DMSO** | **Estimate** | **Std. Error** | **df** | **t value** | **lower** | **upper** | **Pr(>\|t\|)** | **Bonferroni p-value** | |
| --- | --- | --- | --- | --- | --- | --- | --- | --- | --- | --- | --- | --- |
| 24hr | Without agonist | SRB ratio | S0859 10 | 0.000167 | 0.010515 | 86 | 0.0159 | -0.02074 | 0.021069 | 0.987351 | 1 |  |
| 24hr | Without agonist | SRB ratio | DMA 30 | -0.01919 | 0.010515 | 86 | -1.8251 | -0.04009 | 0.001712 | 0.071452 | 0.285807 |  |
| 24hr | Without agonist | SRB ratio | ARC 10 | -0.0022 | 0.010515 | 86 | -0.2094 | -0.0231 | 0.018701 | 0.834658 | 1 |  |
| **24hr** | **PE (10μM)** | **SRB ratio** | **S0859 10** | **0.048717** | **0.010909** | **86** | **4.4659** | **0.027032** | **0.070403** | **2.41E-05** | **9.64E-05** | ******* |
| 24hr | PE (10μM) | SRB ratio | DMA 30 | 0.005642 | 0.010909 | 86 | 0.5172 | -0.01604 | 0.027327 | 0.606373 | 1 |  |
| **24hr** | **PE (10μM)** | **SRB ratio** | **ARC 10** | **0.031979** | **0.010909** | **86** | **2.9316** | **0.010294** | **0.053665** | **0.00432** | **0.017281** | ***** |
| 48hr | Without PE | SRB ratio | DMA | -0.01875 | 0.011134 | 140 | -1.6845 | -0.04077 | 0.003258 | 0.09432 | 1 |  |
| 48hr | Without agonist | SRB ratio | ARC | -0.00559 | 0.011134 | 140 | -0.502 | -0.0276 | 0.016424 | 0.616484 | 1 |  |
| 48hr | Without agonist | SRB ratio | S0859 1 | 0.034119 | 0.014723 | 140.2 | 2.3173 | 0.00501 | 0.063227 | 0.021934 | 0.526407 |  |
| 48hr | Without agonist | SRB ratio | S0859 3 | 0.023299 | 0.011134 | 140 | 2.0926 | 0.001287 | 0.045312 | 0.038189 | 0.916545 |  |
| 48hr | Without agonist | SRB ratio | S0859 10 | 0.032988 | 0.011134 | 140 | 2.9628 | 0.010976 | 0.055001 | 0.003583 | 0.08599 | . |
| 48hr | Without agonist | SRB ratio | S0859 20 | 0.042209 | 0.014723 | 140.2 | 2.8669 | 0.013101 | 0.071318 | 0.004786 | 0.114858 |  |
| **48hr** | **Without agonist** | **SRB ratio** | **S0859 30** | **0.038369** | **0.011134** | **140** | **3.4461** | **0.016357** | **0.060382** | **0.000751** | **0.018025** | ***** |
| 48hr | Without agonist | SRB ratio | S0859 100 | -0.00058 | 0.011134 | 140 | -0.0517 | -0.02259 | 0.021436 | 0.95881 | 1 |  |
| **48hr** | **PE (10μM)** | **SRB ratio** | **DMA 30** | **0.075428** | **0.013351** | **140** | **5.6497** | **0.049032** | **0.101823** | **8.66E-08** | **2.08E-06** | ******* |
| 48hr | PE (10μM) | SRB ratio | ARC | -0.00365 | 0.013351 | 140 | -0.2731 | -0.03004 | 0.022749 | 0.785163 | 1 |  |
| 48hr | PE (10μM) | SRB ratio | S0859 1 | 0.019037 | 0.017655 | 140.2 | 1.0783 | -0.01587 | 0.05394 | 0.282766 | 1 |  |
| 48hr | PE (10μM) | SRB ratio | S0859 3 | 0.022526 | 0.013351 | 140 | 1.6873 | -0.00387 | 0.048921 | 0.093781 | 1 |  |
| **48hr** | **PE (10μM)** | **SRB ratio** | **S0859 10** | **0.131198** | **0.013351** | **140** | **9.827** | **0.104803** | **0.157593** | **1.18E-17** | **2.82E-16** | ******* |
| **48hr** | **PE (10μM)** | **SRB ratio** | **S0859 20** | **0.216517** | **0.017655** | **140.2** | **12.264** | **0.181613** | **0.251421** | **5.99E-24** | **0** | ******* |
| **48hr** | **PE (10μM)** | **SRB ratio** | **S0859 30** | **0.239575** | **0.013351** | **140** | **17.9447** | **0.21318** | **0.26597** | **4.06E-38** | **0** | ******* |
| **48hr** | **PE (10μM)** | **SRB ratio** | **S0859 100** | **0.298129** | **0.013351** | **140** | **22.3304** | **0.271734** | **0.324524** | **5.52E-48** | **0** | ******* |

**Supplementary Methods**

*Cell isolation and culture.* Primary neonatal rat ventricular myocytes were isolated from 1-2 day old Sprague-Dawley rats. Animals were euthanized by cervical dislocation according to Schedule 1 of the UK Home Office regulations. Hearts were minced and serially digested with collagenase and pancreatin. Cell pellets were re-suspended in M1 media (67.5% High Glucose DMEM with glutamine, 5% CO_2_/22 mM NaHCO_3_, 17.5% M199, 10% horse serum, 5% new born calf serum, 100 U Pen/Strep). To separate the myocytes from non-myocytes (e.g. fibroblasts), the cell suspension was placed in culture dishes at 37 °C for 2 hours. This pre-plating step removes non-myocytes on the basis of their stronger adherence to the dish. After 2 hours, media containing unattached cells (mostly myocytes) were collected and cultured for 24 hours on fibronectin-coated chambers (4-chamber Ibidi, Germany) or 96-well plates (black 15 µ-Plate 96 Well, Ibidi, Germany) for imaging, or on 60 mm Petri dishes for extracting mRNA. Cells were then cultured in M2 media (79% High Glucose DMEM with glutamine, 5% CO_2_/22 mM NaHCO_3_, 20% M199, 1% Insulin-Transferring-Selenium-X Supplement and 100 U Pen/Strep) for a further 24 hours, prior to chemical stimulation with agonists or inhibitors. At this point, hypertrophic stimuli were applied in M2 media for 24 or 48 hours. Non-myocyte cells that remained adhered to the dish in the pre-plating step were allowed to proliferate for 72 hr to confluency. At this point, the dominant cell type will be the cardiac fibroblasts; these were trypsinized and re-seeded in imaging chambers or 96-well plates.

*Immunofluorescence.* Cells were washed with phosphate buffered saline (PBS) three times before fixing with 4% paraformaldehyde (4% PFA) in PBS for 15 min. Three further PBS washes were performed to remove fixative. Next, cells were permeabilized with Triton-X (0.2% for myocytes, 0.1% for fibroblasts) in PBS for 10 min, and washed thrice in PBS, before non-specific binding sites were blocked with 5% bovine serum albumin (BSA) in PBS for 1 h. To cells were then incubated with specific antibodies against α-actinin (Sigma, A7732) at 1:500 dilution in 1% BSA, against α-smooth muscle actin (Novus Biologicals, NBP2-22120) at 1:100 dilution, against vimentin (Novus Biologicals, NBP1-97672) at 1:200 dilution, or against prolyl 4-hydroxylase (Origene AD5110-1) at 1:100 dilution, overnight at 4°C in a humid chamber. The following day cells were washed thrice with PBS and incubated with goat anti-mouse Alexa Fluor 488, diluted 1:500 in 1% BSA, for 1 h. In the case of NRVMs, cells were washed with PBS three times and incubated with Hoechst-33342 diluted 1:1000 in PBS for 1 min, and washed with PBS and then imaged. Fibroblasts were mounted using ProLong Diamond Antifade mountant (ThermoFisher Scientific, P36962) and a coverslip and then imaged.

*Real-time PCR.* Total RNA was extracted from cultured neonatal rat cardiomyocytes using TRIzol (Invitrogen) and reverse transcription of RNA samples was carried out using SuperScript IV Reverse Transcriptase (Thermo Fisher Scientific) according to manufacturer’s instructions. Quantitative real-time PCR was performed using TaqMan probes (Applied Biosystems) for Nppa (ANP, assay ID Rn00664637_g1), and Nppb (BNP, assay ID Rn00580641_m1). The housekeeping gene Actb (β-actin, assay ID Rn00667869_m1) was measured in each sample for normalisation. Reactions were set up using TaqMan Fast Universal PCR Master Mix (2X), according to manufacturer’s instructions and assays were performed in a ViiA 7 Real-Time PCR System (Applied Biosystems).

*Details of image analysis.* Particle analysis of Hoechst images was performed to identify nuclei based on thresholding. This involved generating a cumulative-sum histogram and taking the threshold greyscale value to be at 80-90% of the maximum (a value that will depend on the quality of images and intensity distribution). To generate a binary mask, pixels below this threshold were assigned a value of zero, and those above or equal to the threshold were set to a value of one. Particle count was performed using standard algorithms. Particles that were below a critical area (likely representing pixel noise) were excluded. Gaussian mixture model fitting analysed the statistical distribution of particle area, identifying those that represent individual nuclei (small and circular particles) of mononucleate cells, a second (smaller) subset of larger but less symmetrical particles representing two abutting nuclei of the same cell. The cell count was the sum of particles in the first and second subsets. Occasionally, Hoechst staining can appear as larger particles due to high confluency, particularly after a period of hypertrophic growth. The number of cells in such a composite particle was taken as the ratio of its area and the mean nuclei size (average of particles belonging to subsets 1 and 2), and the total cell count raised accordingly. The SRB image was offset for background signal, the level of which was determined by constructing a histogram of intensities and fitting a Gaussian to the component of lowest intensity. Pixels of the SRB image were segregated into those corresponding to nuclear and extra-nuclear region, and averaged. Hypertrophy was quantified as the ratio of extra-nuclear to nuclear SRB, normalized to the cell count. An example of a script, written in MATLAB, for image analysis is included in the Supplement.

**Supplementary Figure**


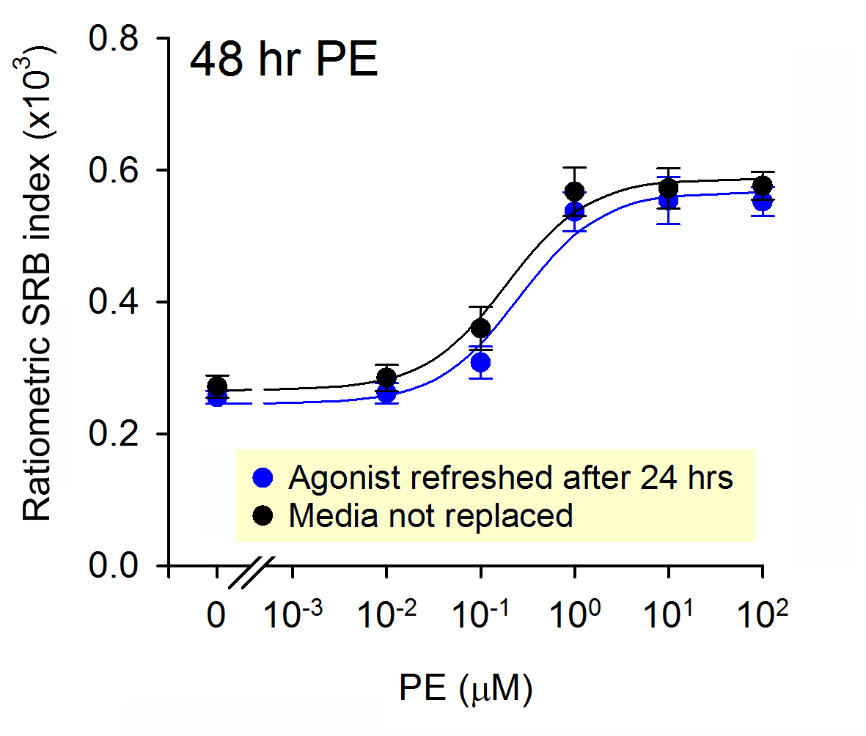


**Figure S1:** NRVM monolayers were treated with various doses of PE, and ratiometric SRB index of growth was reported after 48 hours PE stimulation. In paired experiments, media were replaced at the midpoint (24 hrs) with freshly prepared agonist to test if there is any degree of agonist degradation over the 48 hour incubation period. The results indicate the same dose-dependence, indicating that the agonist stimulation strength was maintained over at least 48 hours in culture. Ascorbate (100 µM) was present in all media to avoid oxidative damage.
